# miR-10a overexpression aggravates renal ischemia–reperfusion injury associated with decreased PIK3CA expression

**DOI:** 10.1186/s12882-020-01898-3

**Published:** 2020-07-01

**Authors:** Dongsheng Xu, Wenjun Li, Tao Zhang, Gang Wang

**Affiliations:** 1 Department of Kidney Transplantation, The Second Hospital of Shandong University, Jinan, 250033 China; 2 Departments of Thoracic Surgery, The Affiliated Yantai Yuhuangding Hospital of Qingdao University, Yantai, 264000 China; 3 Department of Cardiac Surgery, Qingdao Fuwai Cardiovascular Hospital, No. 201 Nanjing Road, Shibei District, Qingdao, 266034 China

**Keywords:** miR-10a, Renal ischemia–reperfusion, Hypoxia–reoxygenation, PIK3CA/PI3K/Akt pathway

## Abstract

**Background:**

To investigate the effect of miR-10a on renal tissues with ischemia reperfusion (I/R) injury in rats and to explore the underlying mechanisms of the effect of miR-10a on hypoxia–reoxygenation in HK-2 cells.

**Methods:**

MiR-10a level was measured in the renal tissues of rats with I/R rats using RT-PCR. In order to research the role of miR-10a in renal tissues, an miR-10 agonist and an miR-10a antagonist were used to treat I/R-injured rats. Levels of serum creatinine and blood urea nitrogen, renal histopathology, and levels of cell apoptosis were analyzed separately in renal tissues from the rats. Phosphatidylinositol 3-kinase (PI3K)/Akt pathway related proteins were measured by Western blotting. In addition, HK-2 cells were cultured in order to study the mechanism of action of miR-10a in the hypoxia-reoxygenation model being studied. Finally, the dual luciferase reporter gene assay was used to confirm that the PI3K p100 catalytic subunit α (PIK3CA) gene was targeted by miR-10a.

**Results:**

After renal I/R injury in rats, miR-10a expression increased significantly (*p* < 0.05). Injection of miR-10a agonist significantly aggravated the renal injury and raised the level of cell apoptosis in the renal tissues of I/R-injured rats (*p* < 0.05). However, administration of miR-10a antagonist led to obvious improvement of the renal injury, decreased renal cell apoptosis, and inhibited PI3K/Akt pathway activity (*p* < 0.05). In in vitro experiments, the negative relationship between PIK3CA and miR-10a levels was confirmed. Furthermore, overexpression of miR-10a significantly decreased the proliferation of HK-2 cells, and increased cell apoptosis via up-regulation of the PI3K/Akt pathway (*p* < 0.05).

**Conclusion:**

The aggravation of renal I/R injury by miR-10a was associated with a decrease in the activity of PIK3CA/PI3K/Akt pathway.

## Background

Renal ischemia–reperfusion (I/R) injury is a common result of complications from certain renal surgeries, such as sepsis, ischemia, and nephrotoxic damage [1]. Renal I/R injury, causes cell homeostasis to be destroyed, leading to inflammation and apoptosis [2]. Furthermore, renal I/R injury can also increase the incidences of other diseases, such as myocardial infarction and stroke [3].

Numerous microRNAs (miRNAs) are known to play critical roles in the pathogenesis renal I/R injury in rats [4]. As a member of the miR-10 family, miR-10a plays essential roles in the process of programmed cell death [5] and in many diseases [6]. However, the potential mechanism by which miR-10a affects renal I/R injury has not yet been reported.

As is widely known, the phosphatidylinositol 3-kinase (PI3K)/Akt pathway is involved in physiological processes underlying a variety of biological reactions, including inflammation and apoptosis [7, 8]. As a target gene of miR-10a, the PI3K p100 catalytic subunit α (PIK3CA) plays a critical role in the cisplatin resistance of lung adenocarcinoma circulating tumor cells via the in PI3K/Akt pathway [9]. Many studies have reported that hypoxia–reoxygenation (H/R) injury can activate the PI3K/Akt pathway to protect renal tubular epithelial cells [10–12]. However, the association between miR-10a and PIK3CA in renal I/R injury has not yet been clarified.

In the present work, the role of miR-10a was investigated in rats with renal I/R injury and in HK-2 cells with H/R damage. Furthermore, the effect of miR-10a on the PIK3CA/PI3K/Akt pathway was also explored in vitro.

## Methods

### Experimental animals

Forty-eight male Sprague-Dawley rats, weighing 220 ± 20 g, were kept at 22–24 °C, and a humidity of 50–60%, under a 12-h dark/light cycle with free access to food and water. All experiments were performed in strict conformity with the National Institutes of Health (NIH) Guidelines pub. No. 85–23, revised 1996 and were approved by the Institutional Animal Care and Use Committee of the Second Hospital of Shandong University (no. 20190103–002).

### Establishment of renal I/R model

Rats were anesthetized by intraperitoneal injection of 3% sodium pentobarbital (50 mg/kg). According to previous reports [13, 14], the renal pedicles were exposed and then the right kidney was removed. The left renal pedicle was clamped for 45 min followed by reperfusion for 24 h. Rats in the sham group underwent a similar operation except that the occlusion of renal pedicle was not performed. After operation, the rats were given free access to food and water.

### Animal groups

Thirty-six rats were randomly divided into 6 groups, 6 in each group. Briefly, the rats were numbered by weight. Then starting from any number in the random number table, 36 numbers were selected from top to bottom and arranged according to the animal number. Odd numbers were used in 1), 3), 5) groups, and even numbers were used in 2), 4), 6) groups. 1) the sham group, in which the rats were injected with normal saline via the tail vein, 1 h before surgery; 2) the renal ischemia–reperfusion group (I/R), in which the rats were subjected to renal ischemia; 3) the miR-10a agonist group (miR-10a), in which the rats were administered 10 mg/kg miR-10a agonist (Ribio, Guangzhou, China) via tail vein injection, 1 h before I/R induction; 4) the miR-10a agonist negative control group (miR-NC), in which the rats were administered 10 mg/kg miR-10a agonist negative control (vehicle) via tail vein injection, 1 h before I/R induction; 5) the miR-10a antagonist group (anti-miR), in which the rats were administered 10 mg/kg miR-10a antagonist (Ribio) via tail vein injection, 1 h before I/R induction; 6) the miR-10a antagonist negative control group (anti-NC), in which the rats were administered 10 mg/kg miR-10a antagonist negative control (vehicle) through tail vein injection, 1 h before I/R induction.

### Sample collection

According to Laboratory animal - Guideline for euthanasia (T/CALAS 31–2017, China), 3% sodium pentobarbital (150 mg/kg), as euthanasia dose, was used to sacrificed rats by intrapertoneal injection after 24 h reperfusion. Five minutes without heartbeat means death. Blood samples (5 mL) were taken from the abdominal aorta. Partial kidneys were stored at − 80 °C and another partially placed in 4% paraformaldehyde for 24 h, then embedded in paraffin.

### Real-time polymerase chain reaction (RT-PCR)

Total RNA samples were extracted using a TRIzol Kit (ThermoFisher, Guangzhou, China). The cDNA was synthesized using the TaqMan Reverse Transcription Kit (4,366,596; ThermoFisher) under the following conditions: 95 °C for 15 s, 60 °C for 30 s, and 72 °C for 45 s (50 cycles). The 2^-ΔΔCt^ method was used to analyze the data.

The following primers were used: miR-10a-5′, CTGGAAAATTTCTGGGCCAA; miR-10a-3′, CCAGACTGTCCTCATTCAGAAAAA; U6–5′, GACCTCTATGCCAACACAGT; U6–3′, AGTACTTGCGCTCAGGAGGA. PIK3CA-5′, GCATACATTCGAAAGACC; PIK3CA-3′, CTCAGTTATCTTTTCAG; GAPDH-5′, TGACTTCAACAGCGACACCCA; GAPDH-3′, CACCCTGTTGCTGTAGCCAAA.

### Renal function test

Blood samples were collected and centrifuged at 800×g for 10 min. The levels of serum creatinine (Scr) and blood urea nitrogen (BUN) in serum were analyzed using an automatic biochemical.

### Hematoxylin-eosin (H&E) staining

Renal sections (5 μm) were stained with H&E (Solarbio, Wuhan, China) and histopathological changes were observed at 400× magnification. The swelling, vacuolation, and exfoliation of renal tubular epithelial cells were evaluated using a five-point quantitative scoring method [15]: 0, < 10%; 1, 10–25%; 2, 25–50%; 3, 50–75%; and 4, 75–100%.

### Terminal dexynucleotidyl transferase (TdT)-mediated dUTP nick end labeling (TUNEL)

Levels of cell apoptosis in renal tissue sections (5 μm) were measured using a TUNEL Apoptosis Assay Kit (Solarbio) with sections randomly observed at 400× magnification to identify apoptotic nuclei stained yellow brown or dark brown. The apoptotic index (AI) was calculated as: = (number of apoptotic cells/total cells) × 100%.

### Western blottiong

The concentration of protein in the sample was measured using a BCA Protein Quantification Kit (Solarbio, Beijing, China). Forty micrograms samples were mixed with 10% SDS-PAGE before being transferred to PVDF membranes (Millipore, Massachusetts, USA). The membranes were blocked with 5% degreased milk powder for 1 h. The primary antibody of each protein was diluted with 5% BSA as follows: rabbit anti-rat Bax(1:800, orb224426, Biorbyt, Cambridge, UK), Bcl-2(1:800, orb228150, Biorbyt), caspase-3(1:800, orb10231, Biorbyt), PIK3CA (1:800, orb228203, Biorbyt), PI3K (1:800, orb137259, Biorbyt), p-PI3K (1:800, orb338965, Biorbyt), Akt (1:800, orb213545, Biorbyt), p-Akt (1:800, orb222951, Biorbyt), and β-actin (1:2000, orb178392, Biorbyt). All primary antibodies were reacted overnight at 4 °C, then incubated with the secondary goat anti-rabbit Ig G (1:1500, ab6721, Abcam, Beijing, China) for 1 h.

### Cell culture

HK-2 cells (Shanghai Institute of Cell Research, Shanghai, China), which are human renal tubular epithelial cells, were cultured in DMEM/F12 with 10% fetal bovine serum and 1% penicillin-streptomycin (GIBCO, Invitrogen, Beijing, China) at 37 °C, 5% CO_2_.

### Establishment of H/R model

The hypoxia induced cell injury model was established as follows: HK-2 cells were cultured in an anoxic environment (5% CO_2_, 1% O_2_, 94% N_2_) for 24 h, then transferred to an aerobic environment (5% CO_2_, 21% O_2_, 74% N_2_) for 3 h.

### Cell transfection

Before transfection, cells were plated into a six-well plate for 24 h. Lipofectamine TM2000 (Invitrogen, Carlsbad, CA, USA) and plasmid (GenePharma, Shanghai, China) were separately added to sterile centrifuge tubes containing 200 μL DMEM/F12 for 5 min. Then, the two solutions were mixed for 20 min. Finally, the cells were cultured in six-well plates for 6 h. Six groups were assigned according to the different treatment: 1) the control group, in which cells were cultured under normal condition for 27 h; 2) the hypoxia/reoxygenation group (H/R), in which cell injury was induced by hypoxia; 3) the miR-10a mimic group (miR-10a), in which cells were transfected with miR-10a mimics (5′-CAAAUUCGGAUCUACAGGGUAUU-3′ and anti-5′-UACCCUGUAGAUCCGAAUUUGUG-3) 6 h before H/R induction; 4) the miR-10a mimic negative control group (miR-NC), in which cells were transfected with miR-10a mimic negative control (5′-UUCUCCGAACGUGUCACGUTT-3′ and anti-5′-UACCCUGUAGAUCCGAAUUUGUG-3′) 6 h before H/R induction; 5) the miR-10a inhibitor group (anti-miR), in which cells were transfected with miR-10a inhibitor (5′-CACAAAUUCGGAUCUACAGGGUA-3′) 6 h before H/R induction; 6) miR-10a inhibitor negative control group (anti-NC), in which cells were transfected with miR-10a inhibitor negative control (5′-CAGUACUUUUGUGUAGUACAA-3′) 6 h before H/R induction.

### CCK-8

Cells (2 × 10^4^ cells/mL) were cultured at 37 °C, 5% CO_2_ for 24 h, 48 h, 72 h, and 96 h. Ten microliters CCK-8 solution (Beyotime, Wuhan, China) was added into each well for 4 h. The absorbance (OD) value measured at 450 nm.

### Clone formation

Two milliliters cells (250 cells/mL) were planted into six-well plate, then cultured at 37 °C, 5% CO_2_ for 2 weeks. And the cell medium was freshly changed every 3 days. Methanol was used to fix the cells and 1 mL Giemsa solution (Beyotime) was used to stain them for 35 min, then washed twice with ultrapure water.

### Flow cytometry

After culturing 24 h, cells were collected and washed with pre-cooled 1 × PBS at 4 °C. The cells were suspended in 200 μL of 1× binding buffer and then 5 μL FITC was added for 15 min at 37 °C for labeling. Before cytometric analysis, 150 μL of 1 × binding buffer was added to 5 μL propidium iodide (PI) for cell staining. The cells were placed in a flow cytometer and analyzed using Cell Quest software.

### Dual luciferase reporter assay

The correlation of miR-10a and PIK3CA was predicted by RegRNA 2.0. miR-10a mimics or control scrambled sequences were transfected into HK-2 cells for 48 h using Lipofectamine 2000 at a 2:1 M ratio. The activity of luciferase in cells was tested by the dual-luciferase assay (Solarbio, Wuhan, China).

### Statistical analysis

Data analysis was performed by SPSS 19.0, and the results were represented as mean ± standard deviation (x̄ ± SD). The t-test was used to compare two groups, while the one-way analysis of variance and Tukey’s test were used to compare multiple groups. *p* < 0.05 was considered to indicate a statistically significant difference.

## Results

### miR-10a is overexpressed in renal I/R rats

**Fig. 1:**
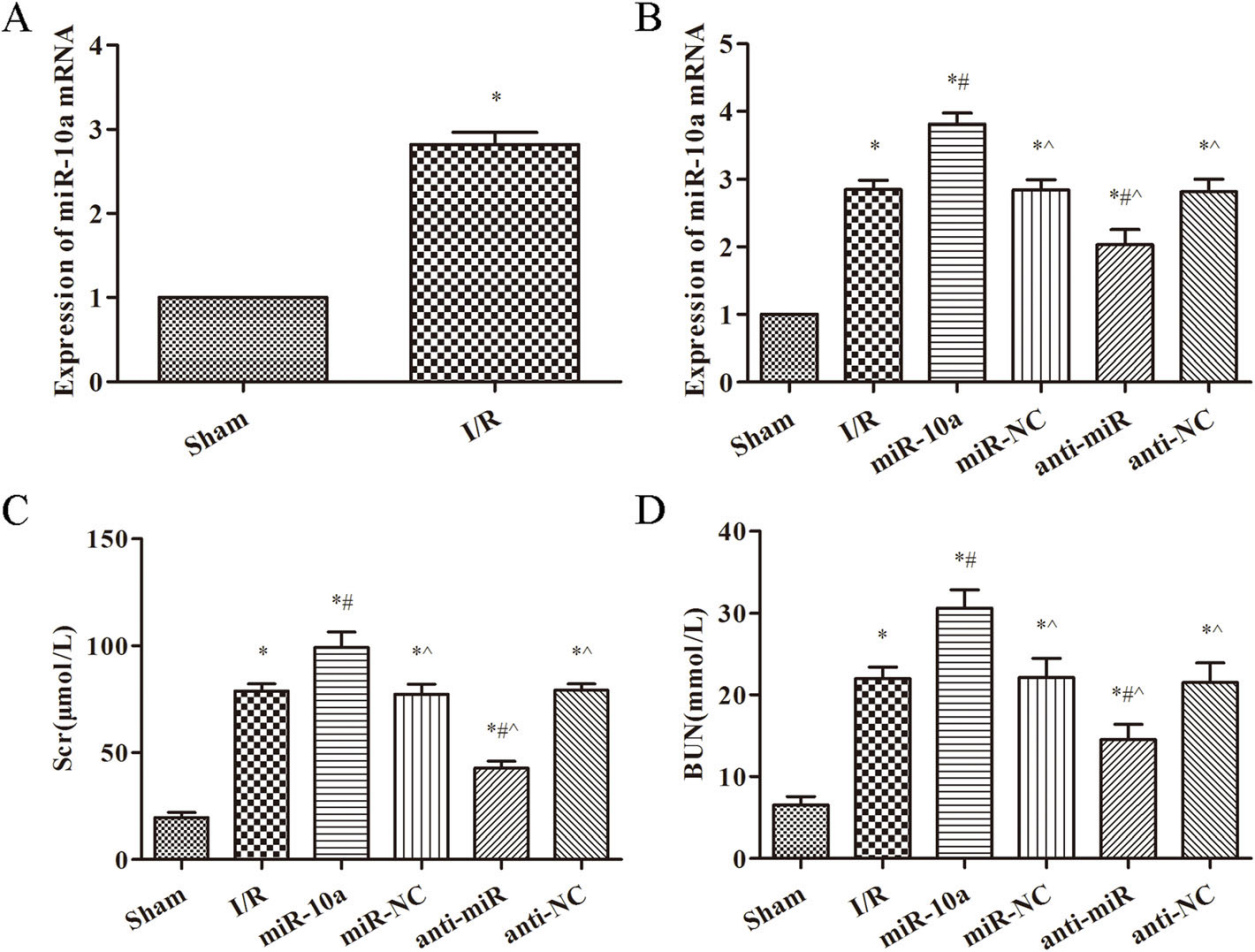
miR-10a expression was increased in kidney tissues with renal I/R injury rats and its overexpression aggravated the renal function in renal I/R rats. **a**, **b** RT-PCR was used to detect the miR-10a expression in kidney tissue; **c**, **d** Serum creatinine (Scr) and urea nitrogen (BUN) levels were measured in serum. Renal ischemia-reperfusion group (I/R), miR-10a agonist group (miR-10a), miR-10a agonist negative control group (miR-NC), miR-10a antagonist group (anti-miR), miR-10a antagonist negative control group (anti-NC). *p < 0.05 compared to Sham group; #*p* < 0.05 compared to I/R group; ^*p* < 0.05 compared to the miR-10a group (*n* = 6)

As showed in Fig. 1a, the expression of miR-10a was clearly increased in the I/R group compared with the sham group (*p* < 0.05). RT-PCR was used to detect the expression of miR-10a in each group, the results are showed in Fig. 1b. Among all the groups, expression of miR-10a was the highest in the miR-10a group and the lowest in the sham group (*p* < 0.05). Furthermore, the Scr and BUN levels were significantly increased after renal I/R injury when compared to the sham group (*p* < 0.05, Fig. 1c, d). Compared with the I/R group, levels of Scr and BUN were obviously raised in the miR-10a group, and notably decreased in the anti-miR group (*p* < 0.05, Fig. 1c, d).

### miR-10a overexpression aggravates renal injury in renal I/R rats

**Fig. 2:**
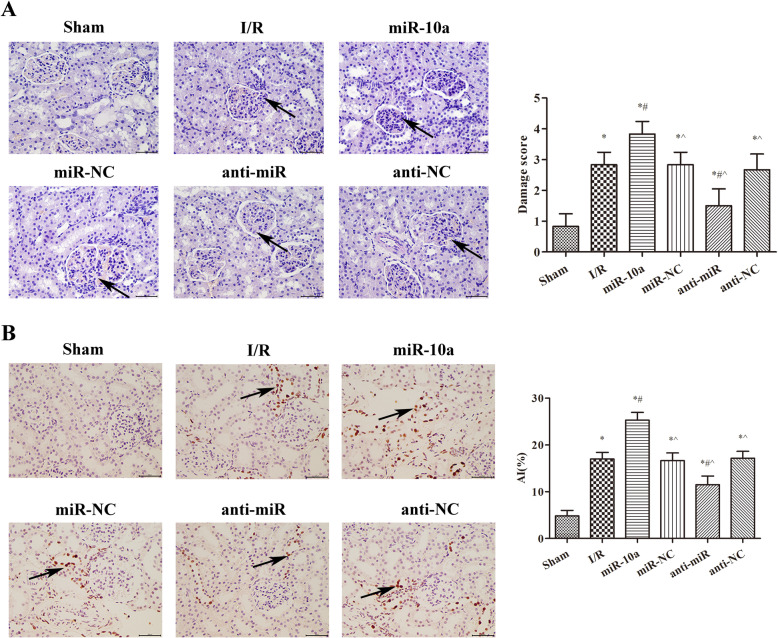
miR-10a overexpression aggravated renal tissue injury and increased cells apoptosis in renal tissues. **a** HE staining was used to observe the kidney tissue pathological changes. **b** TUNEL staining was used to observe the apoptosis of renal cells. *p < 0.05 compared to Sham group; #*p* < 0.05 compared to I/R group; ^*p* < 0.05 compared to the miR-10a group (*n* = 6)

As showed in Fig. 2a, the renal injury score significantly increased after renal I/R injury compared to the sham group (*p* < 0.05). Renal injury was further exacerbated in the miR-10a group compared with the I/R group (*p* < 0.05). However, renal injury was notably improved in the anti-miR group compared to the I/R group (*p* < 0.05). The levels of cell apoptosis in renal tissues in each group were showed in Fig. 2b. Among the groups, the apoptotic index (AI) was the highest in the miR-10a group and the lowest in the sham group (*p* < 0.05). Compared with the I/R group, the AI was notably decreased in the anti-miR group (*p* < 0.05). These data suggest that miR-10a overexpression aggravates renal injury in renal I/R rats.

### miR-10a overexpression inhibited the PIK3CA/PI3K/Akt pathway

**Fig. 3:**
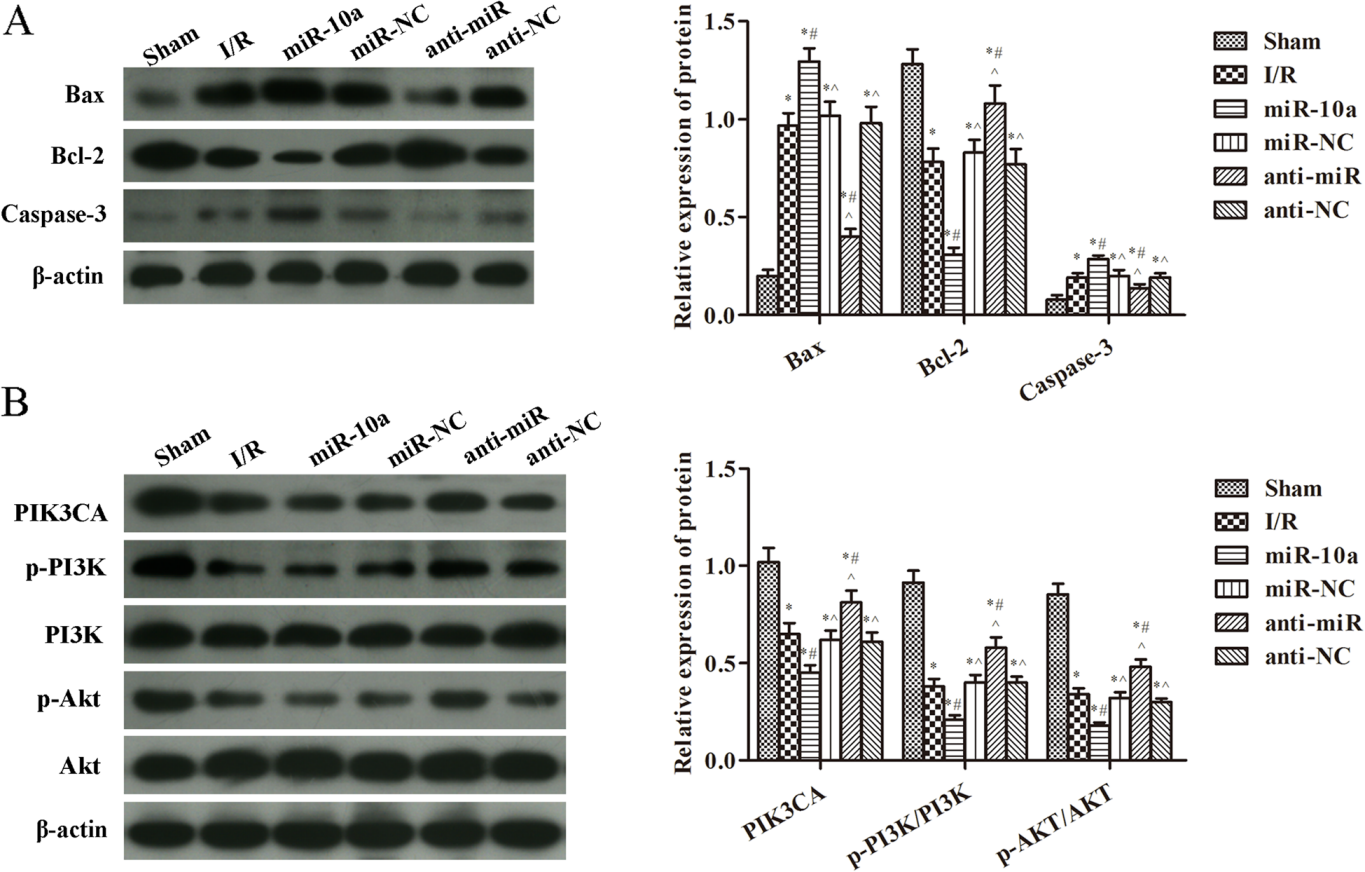
miR-10a overexpression increased apoptosis related proteins and inhibited PIK3CA/PI3K/Akt pathway. **a** Western blot was used to detect the expression of Bax, Bcl-2 and Caspase-3 in renal tissues; **b** Western blot was used to detect the expression of PIK3CA, p-PI3K/PI3K, p-Akt/Akt in renal tissues. *p < 0.05 compared to Sham group; #*p* < 0.05 compared to I/R group; ^*p* < 0.05 compared to the miR-10a group (*n* = 6)

In Fig. 3a, compared with the sham group, the expression of Bax and caspase-3 was significantly increased, and the expression of Bcl-2 was markedly decreased after I/R injury (*p* < 0.05). Compared with the I/R group, the Bax and caspase-3 expression were further increased, and the Bcl-2 expression was further decreased in the miR-10a group (*p* < 0.05). However, the Bax and caspase-3 expression were notably decreased, and the Bcl-2 expression was significantly increased in the anti-miR group when compared to the I/R group (*p* < 0.05). In Fig. 3b, the PIK3CA, p-PI3K and p-Akt expression was clearer lower after renal I/R injury compared with the sham group (*p* < 0.05). In the miR-10a group, levels of PIK3CA, p-PI3K and p-Akt were further lowered. Nevertheless, the PIK3CA, p-PI3K and p-Akt expression was increased in the anti-miR group contrasted to the I/R group (*p* < 0.05).

### miR-10a overexpression inhibites H/R induced HK-2 cell proliferation

**Fig. 4:**
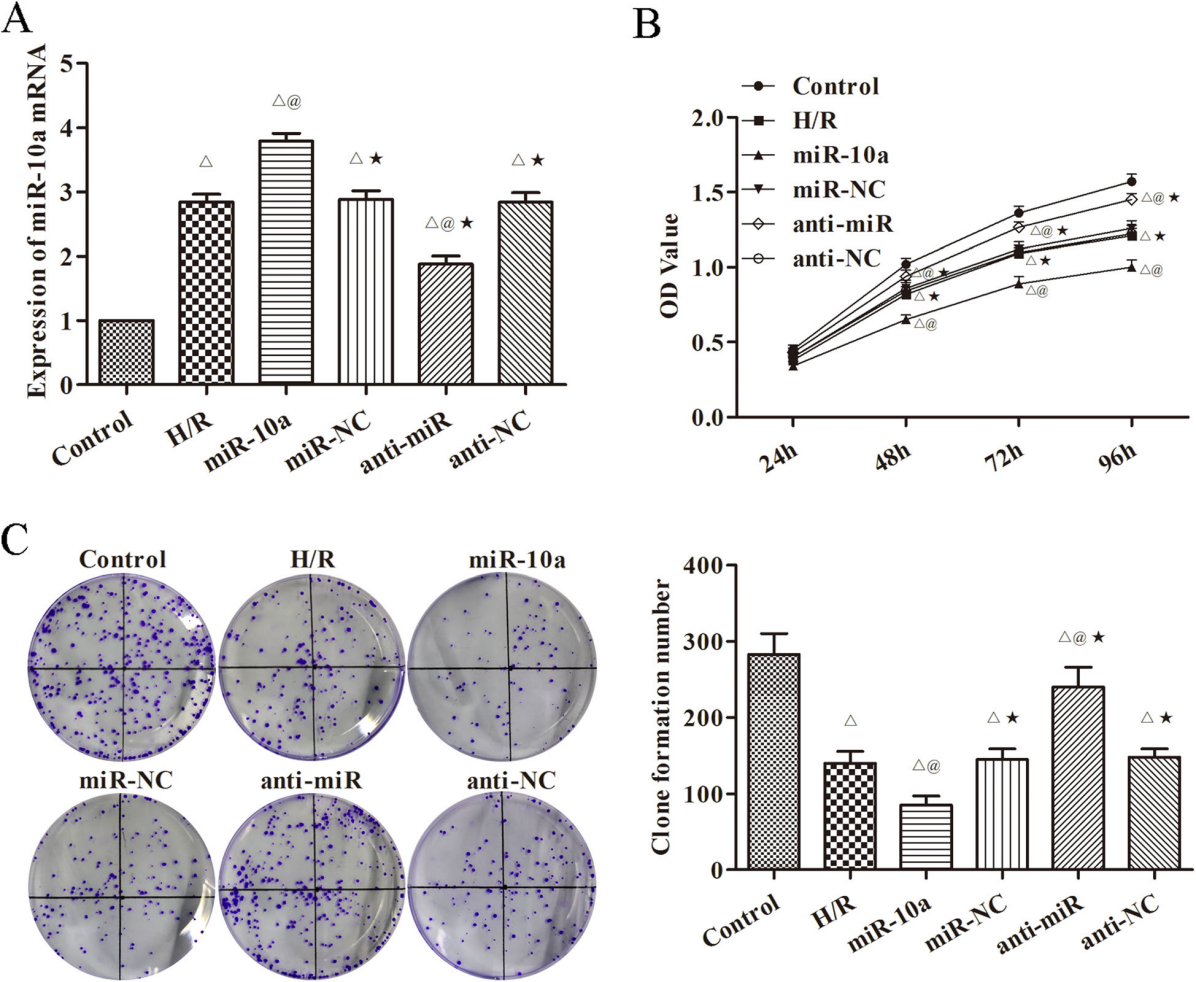
miR-10a overexpression inhibited the proliferation of H/R induced HK-2 cell. **a** RT-PCR was used to detect the expression of miR-10a in cells; **b**, **c** The proliferation of cells in each group was detected by CCK8 and cloning formation experiment. The more clone number, the stronger cell proliferation. Hypoxia/reoxygenation group (H/R), miR-10a mimic group (miR-10a), miR-10a mimic negative control group (miR-NC), miR-10a inhibitor group (anti-miR), miR-10a inhibitor negative control group (anti-NC). ^△^*p* < 0.05 compared to control group; ^@^*p* < 0.05 compared to H/R group; ^★^*p* < 0.05, compared to the miR-10a group (*n* = 6)

In order to confirm that miR-10a overexpression is able to control renal cell proliferation, miR-10a overexpression and underexpression were established in H/R induced HK-2 cells. RT-PCR was used to test the expression of miR-10a in each group (Fig. 4a) and cell proliferation was quantified (Fig. 4b, c). Similar to the in vivo results, cell proliferation was significantly increased in the miR-10a group compared with the H/R group (*p* < 0.05). However, proliferation was significantly decreased in the anti-miR group compared with the H/R group (*p* < 0.05).

### miR-10a overexpression increased H/R induced HK-2 cells apoptosis

**Fig. 5:**
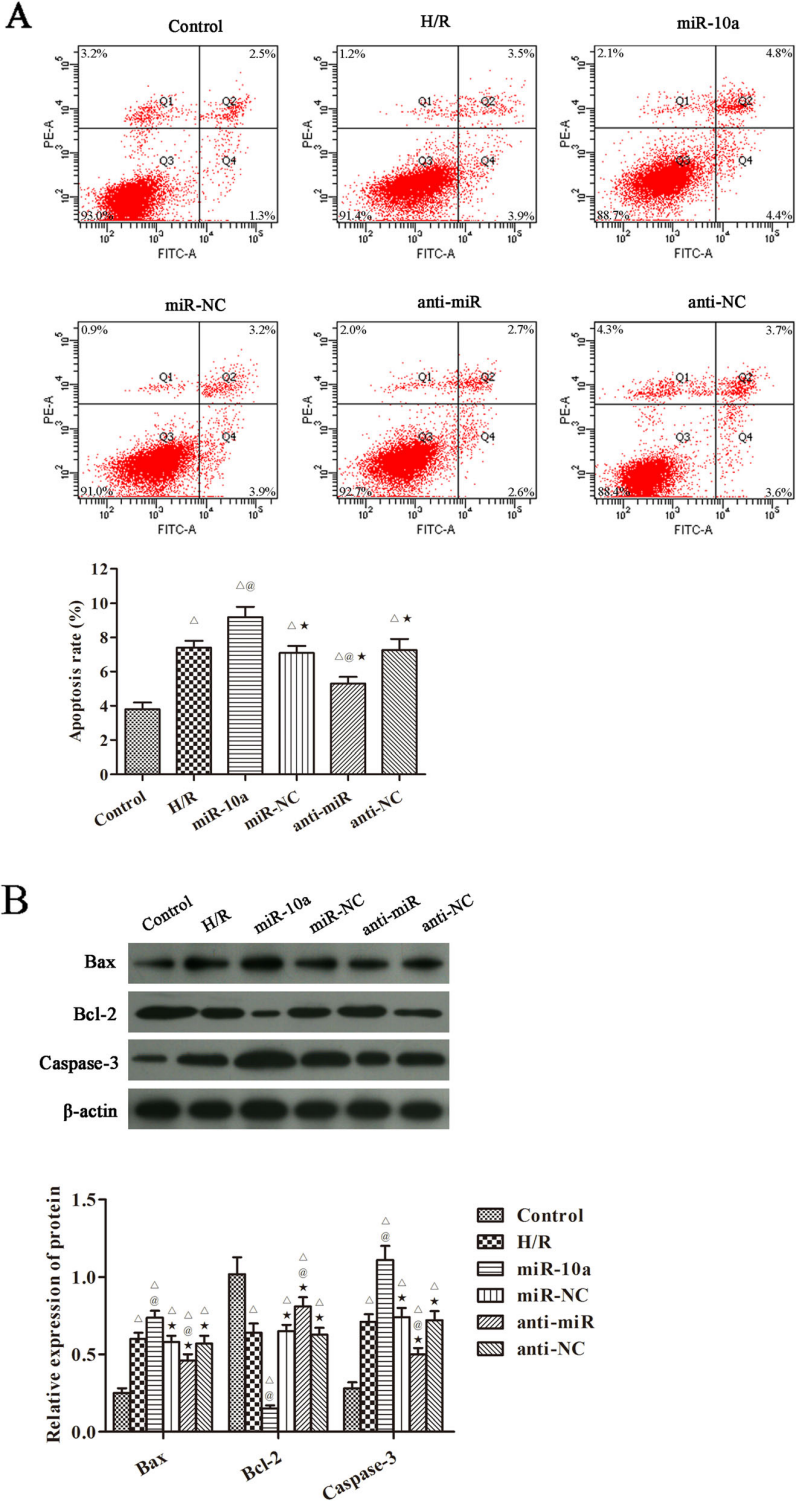
miR-10a overexpression increased cells apoptosis and apoptosis related proteins in H/R induced HK-2 cells. **a** The cells apoptosis was detected by flow cytometry; **b** Western blot was used to detect the expression of Bax, Bcl-2 and Caspase-3 proteins. ^△^*p* < 0.05 compared to control group; ^@^*p* < 0.05 compared to H/R group; ^★^*p* < 0.05, compared to the miR-10a group (*n* = 6)

Levels of HK-2 cell apoptosis were analyzed in Fig. 5a. Among the groups, apoptosis was the highest in the miR-10a group and the lowest in the control group (*p* < 0.05). Compared with the H/R group, the apoptosis was notably decreased in the anti-miR group (*p* < 0.05). In terms of the apoptosis related proteins, the results were consistent with those obtained in vivo (Fig. 5b). Compared with the control group, the expression of Bax and caspase-3 was significantly increased, the expression of Bcl-2 was obviously decreased after H/R injury (*p* < 0.05). Compared with the H/R group, the Bax and caspase-3 expression were further raised, and Bcl-2 expression was further lowered in the miR-10a group (*p* < 0.05). However, the Bax and caspase-3 expression were notably decreased, and the Bcl-2 expression was significantly increased in the anti-miR group when compared to the H/R group (*p* < 0.05).

### miR-10a targetes PIK3CA to regulate PI3K/Akt pathway in H/R induced HK-2 cells

**Fig. 6:**
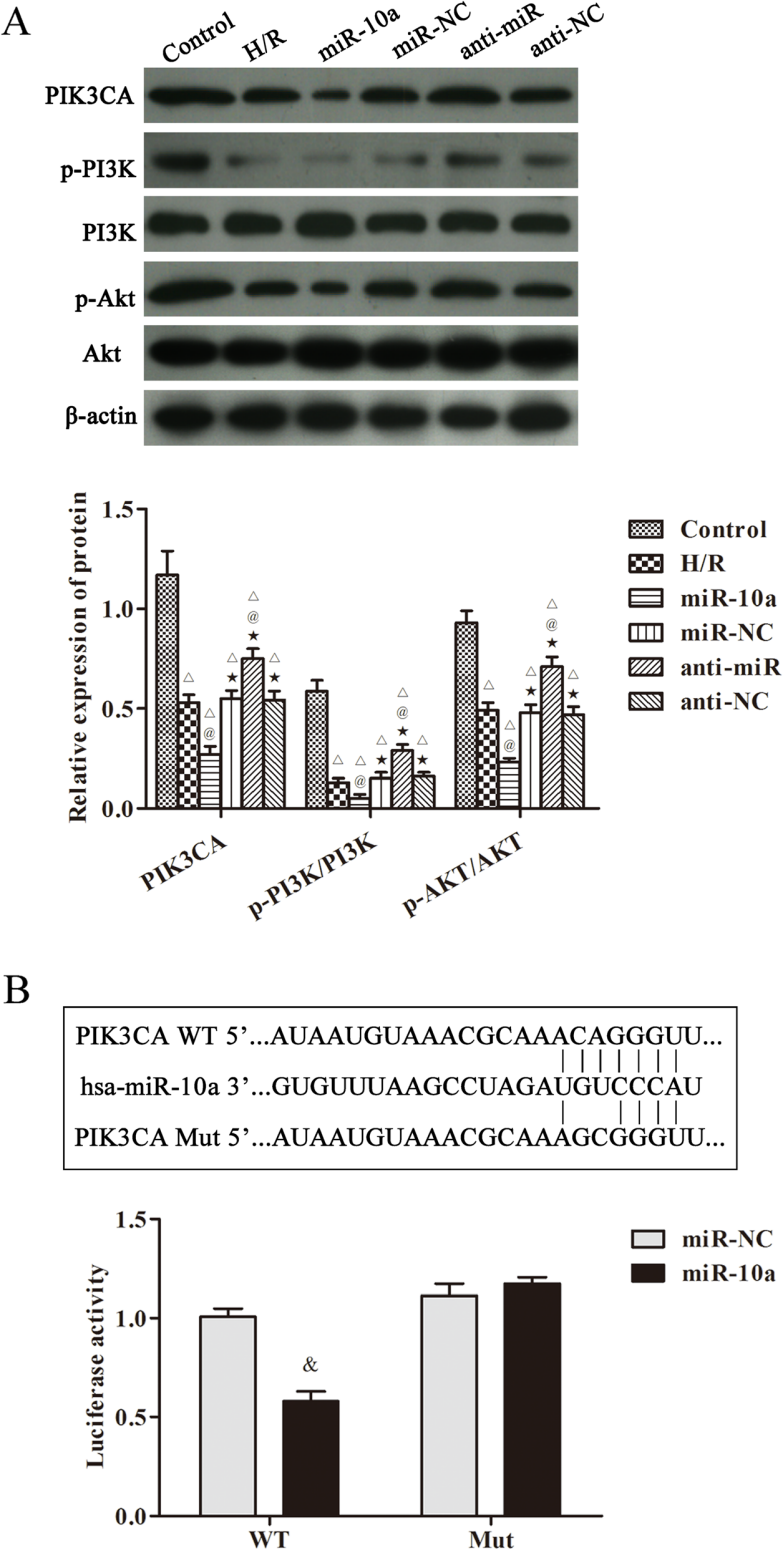
miR-10a overexpression inhibited PIK3CA/PI3K/Akt pathway in H/R induced HK-2 cells. **a** Western blot was used to detect the expression of PIK3CA, p-PI3K/PI3K, p-Akt/Akt in cells; **b** Double luciferase reporter result of recombinant vector of miR-10a and targeted gene PIK3CA. ^△^*p* < 0.05 compared to control group; ^@^*p* < 0.05 compared to H/R group; ^★^*p* < 0.05, compared to the miR-10a group (*n* = 6). ^&^*p* < 0.05 compared to the miR-NC group (*n* = 6)

The PIK3CA/PI3K/Akt pathway related proteins in H/R induced HK-2 cells were also observed in Fig. 6a. The expression of PIK3CA, p-PI3K and p-Akt was clearly declined after H/R injury compared with the control group (*p* < 0.05). In miR-10a group, the expression of PIK3CA, p-PI3K and p-Akt was further decreased. However, the levels of PIK3CA, p-PI3K and p-Akt were clearly increased in the anti-miR group contrasted to the H/R group (*p* < 0.05). Furthermore, a dual luciferase reporter system confirmed that miR-10a targets PIK3CA (Fig. 6b). These data suggest that PIK3CA is a target gene of miR-10a and that miR-10a aggravates renal injury via regulating the PIK3CA/PI3K/Akt pathway.

## Discussion

Current knowledge indicates that renal I/R injury is the primary reason for acute kidney failure and renal damage [16, 17]. Renal I/R injury could lead to autoimmune inflammatory reaction, vascular leakage and promotion of cell death. As indicators of kidney function, Scr and BUN were significantly higher in renal injury. In the present study, the levels of Scr and BUN were notably higher in I/R group than sham group, which indicated that the Renal I/R model was successfully prepared.

Increasing evidences suggest miRNAs play a crucial role in the occurrence and development of diseases. In terms of renal I/R injury, miRNAs play an important role in different aspects of such injury, including apoptosis and inflammation [18, 19]. Xie et al. reported that miR-128-3p improved the acute kidney injury via inhibiting cell apoptosis [18]. Importantly, Shan et al. showed that miR-10a played an important role in extracellular matrix accumulation in the kidneys of diabetes mellitus model animals, in which the level of miR-10a decreased after administration of a high fat diet [20]. Moreover, a further study reported that the level of miR-10a in urine positively correlated with the degree of kidney injury induced by renal I/R. Furthermore, compared to healthy donors, miR-10a levels in urine were substantially elevated in focal segmental glomerulosclerosis patients [21]. In the present study, we found that miR-10a expression was markedly up-regulated in the renal tissues of renal I/R rats. In addition, miR-10a overexpression aggravated the renal injury and cells apoptosis in vivo. This indicated that miR-10a played a negative role in renal I/R injury.

Bcl-2 family members play an important role in apoptosis of renal injury. Bax is one of the Bcl-2 family proteins, which induces cells apoptosis by forming oligomers in the outer membrane of mitochondria to form a transport channel for cytochrome C (CytC) [14]. CytC locates in the cytosol and plays a crucial role in the activation of caspase-3. The increase of caspase-3 and the Bax/Bcl-2 ratio reveals cells apoptosis increase [12]. In our study, renal I/R could induce the release of caspase-3 and the Bax/Bcl-2 ratio, meanwhile, miR-10a overexpression increased the expression of caspase-3 and Bax/Bcl-2 ratio. All the results suggested that miR-10a exacerbated renal I/R injury through promoting renal cells apoptosis.

PIK3CA locates on chromosome 3 and encodes the p110α catalytic subunit of PI3K, which can enhance cell activity and tumour formation via positively regulating PI3K/Akt activity [9]. PIK3CA regulates the generation of phosphatidylinositol triphosphate to activate Akt/PKB kinase. The PI3K/Akt signaling pathway plays an important role in the regulation of cell proliferation and survival, and has been showed to be involved in protecting brain and kidney from I/R injury by reducing oxidative stress and the inflammatory response [18, 22]. Moreover, studies have found that I/R decreased the phosphorylation of PI3K and Akt in renal tissues, and that activation of the PI3K/Akt pathway ameliorates renal I/R injury [11, 23]. Consistently, the PIK3CA and PI3K/Akt pathway were inhibited in rats with renal I/R injury and in H/R cells in this research. Furthermore, miR-10a overexpression further inhibited the PIK3CA and PI3K/Akt pathway, and silence of miR-10a increased the PIK3CA and PI3K/Akt pathway in vivo and in vitro. More importantly, we provided evidence that PIK3CA might act as a target of miR-10a. These data revealed that miR-10a might target PIK3CA to regulate PI3K/AKT signaling pathway in renal I/R injury.

## Conclusions

In summary, the present work explored the increasing expression level of miR-10a during renal I/R injury. Overexpression of miR-10a aggravated renal injury and inhibited PIK3CA/PI3K/Akt pathway in vivo and in vitro*.* These results provide novel insights into the interaction between miR-10a expression and renal I/R injury.

## Supplementary information

**Additional file 1.**

## Data Availability

The figures data used to support the findings of this study were supplied by T Zhang under license and so cannot be made freely available. Requests for access to these data should be made to T Zhang, bgpzopzk@163.com.

## Abbreviations

I/R: Ischemia-reperfusion
Scr: Serum creatinine (Scr)
BUN: Blood urea nitrogen
PI3K: Phosphatidylinositol 3-kinase
PIK3CA: PI3K p100 catalytic subunit
H/R: Hypoxia/reoxygenation
AI: Apoptotic index
