# Supplementary material for: miR-10a overexpression aggravates renal ischemia–reperfusion injury associated with decreased PIK3CA expression
Source: BMC Nephrol. 2020 Jul 1;21:248. doi: 10.1186/s12882-020-01898-3 (PMC7329557; doi:10.1186/s12882-020-01898-3)

**Figure 3**

**A**

Bax:


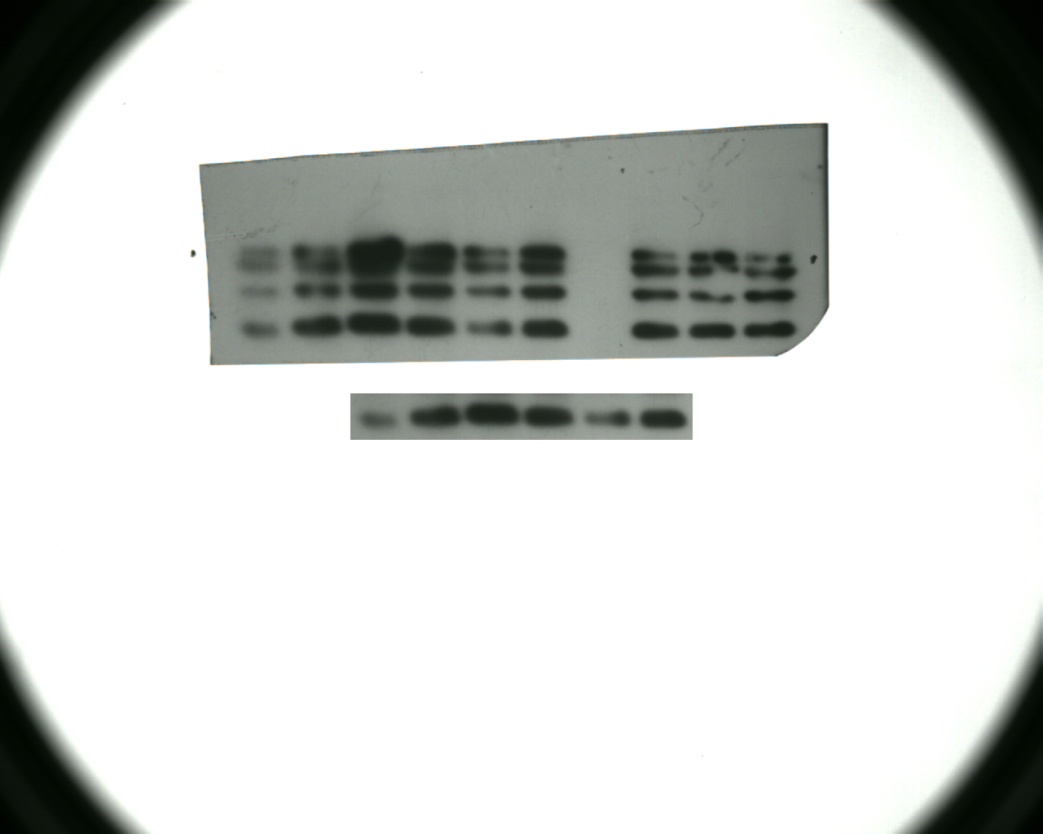


Bcl-2:


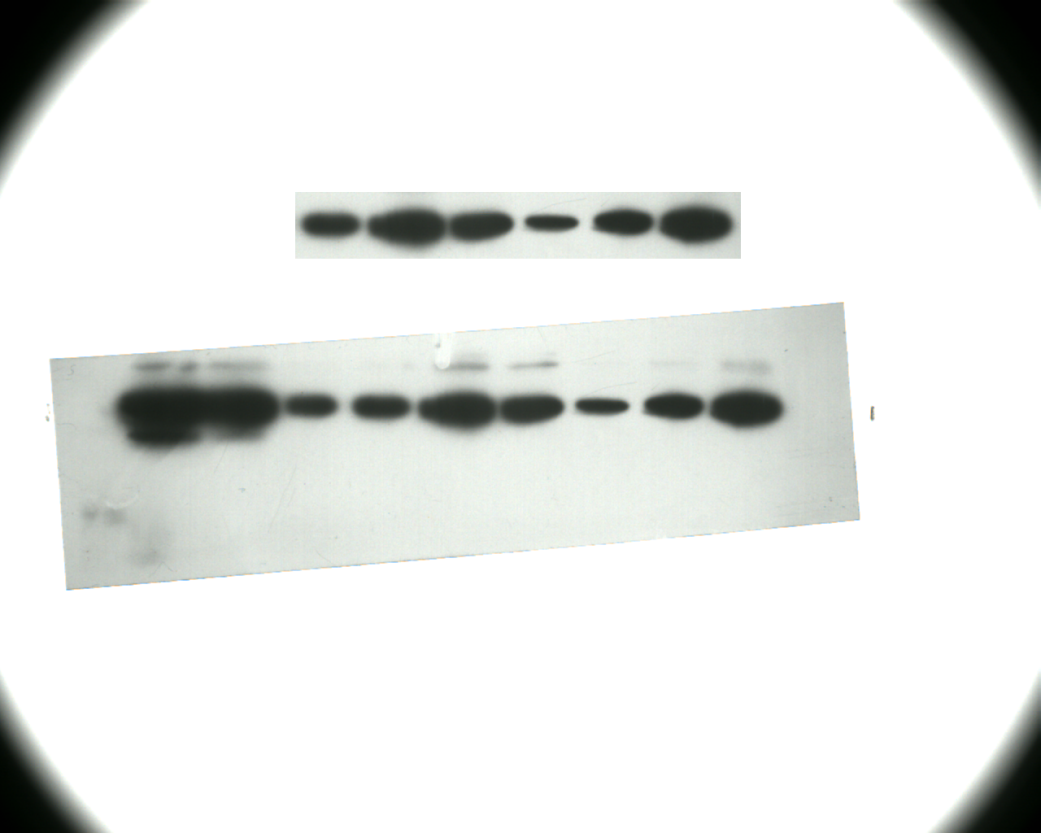


Caspase-3:


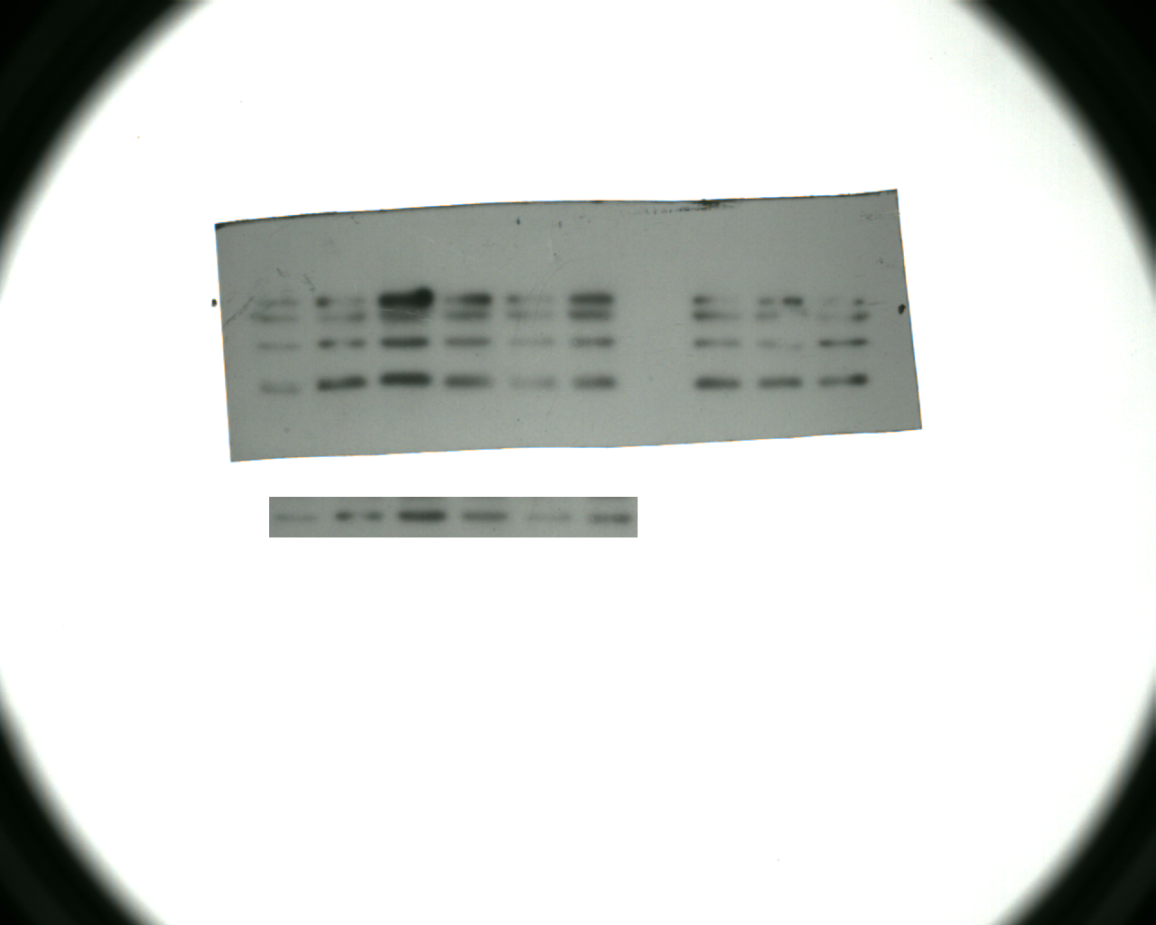


β-actin:


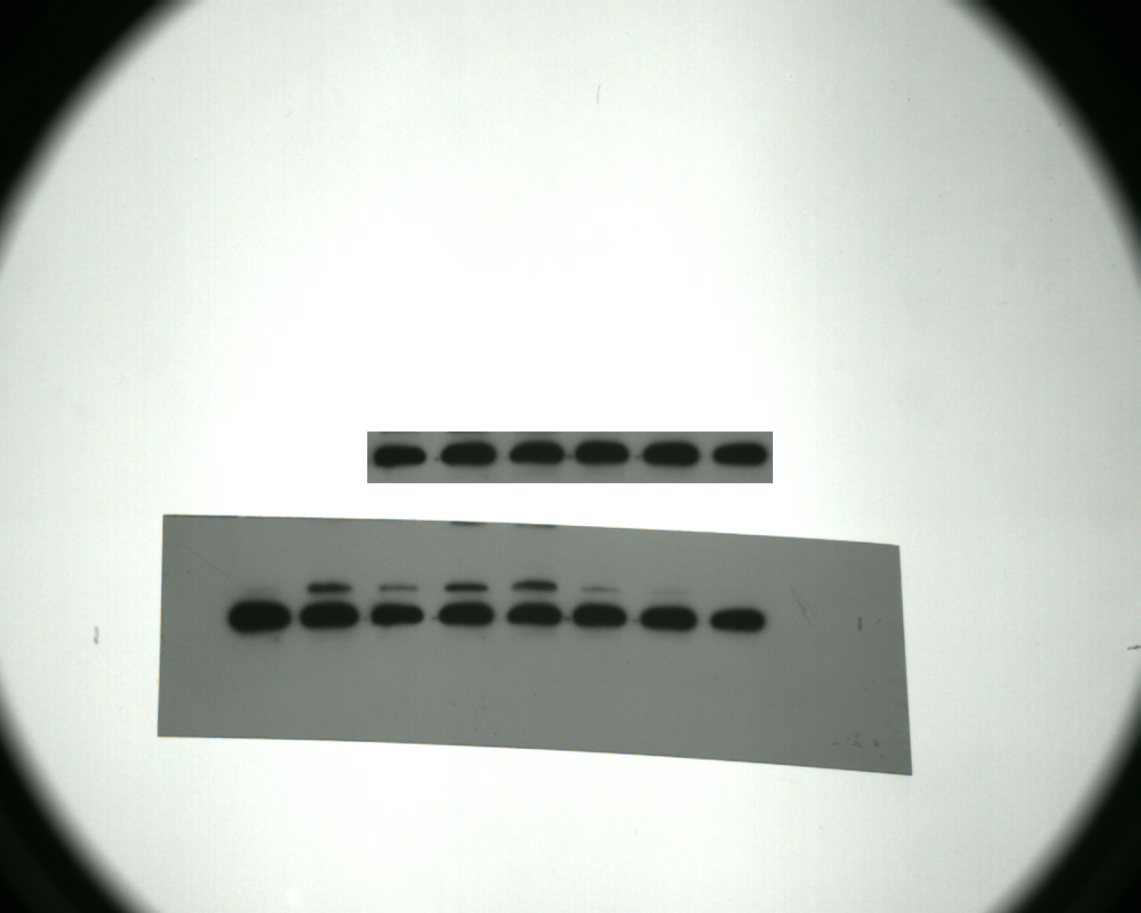


**B**

Akt:


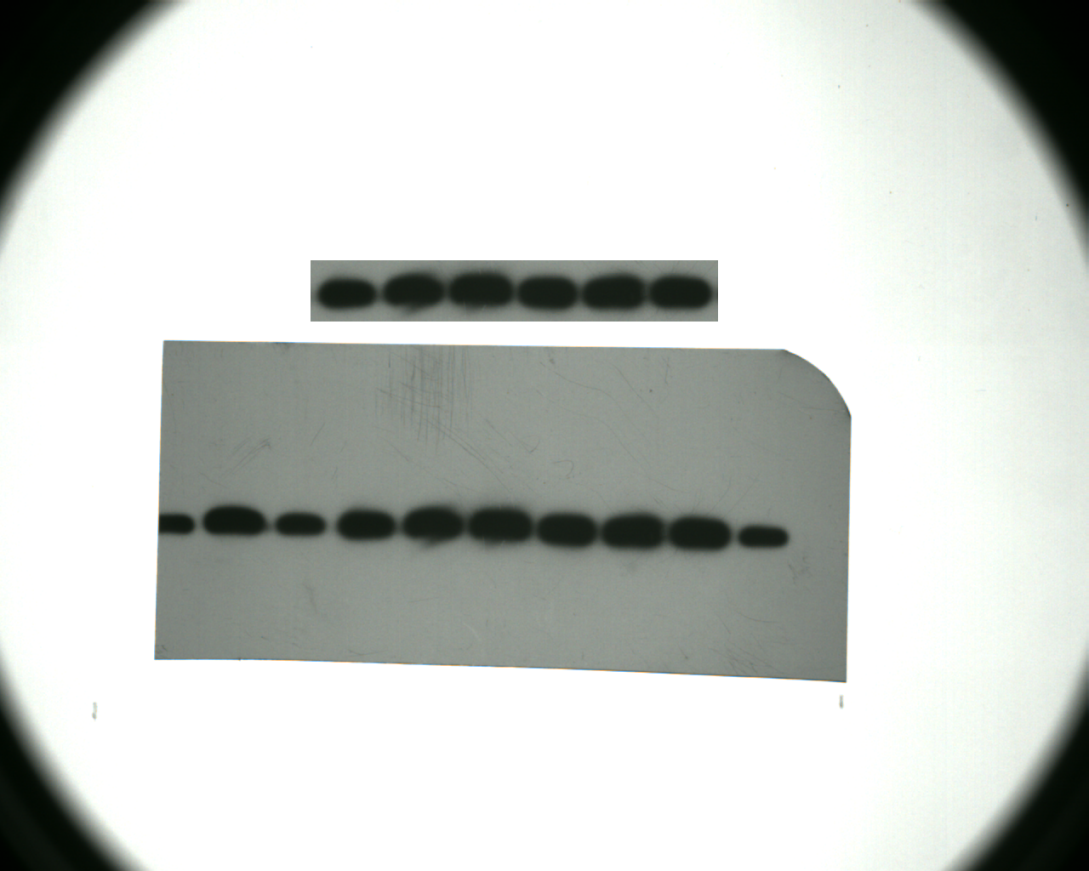


p-Akt:


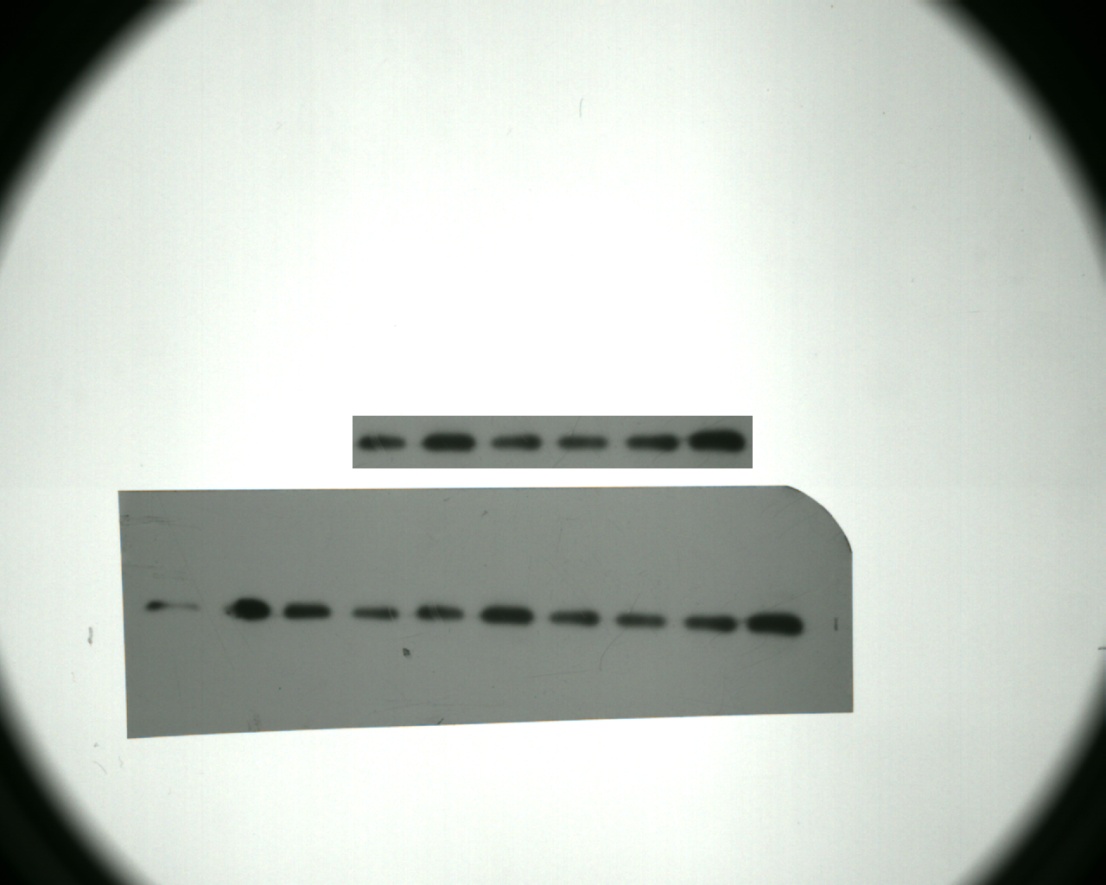


PI3K:


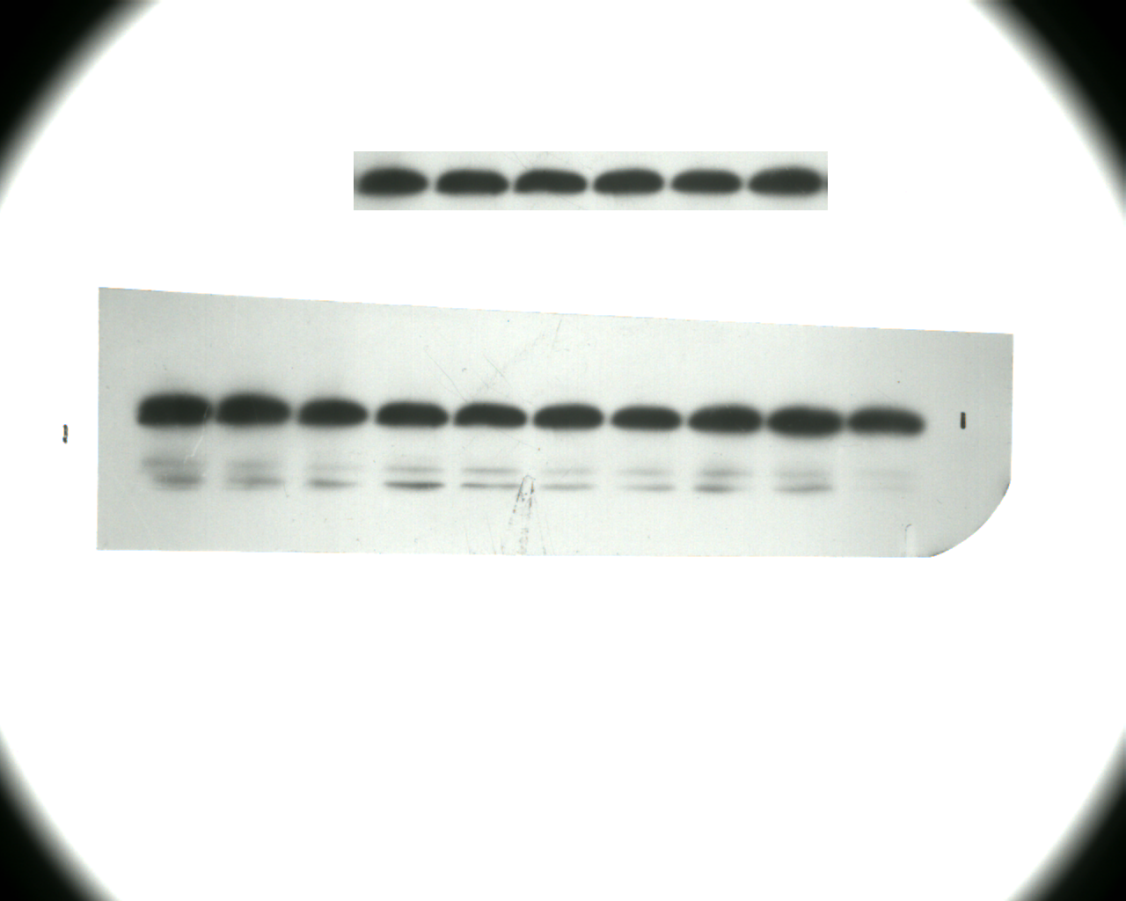


PIK3CA:


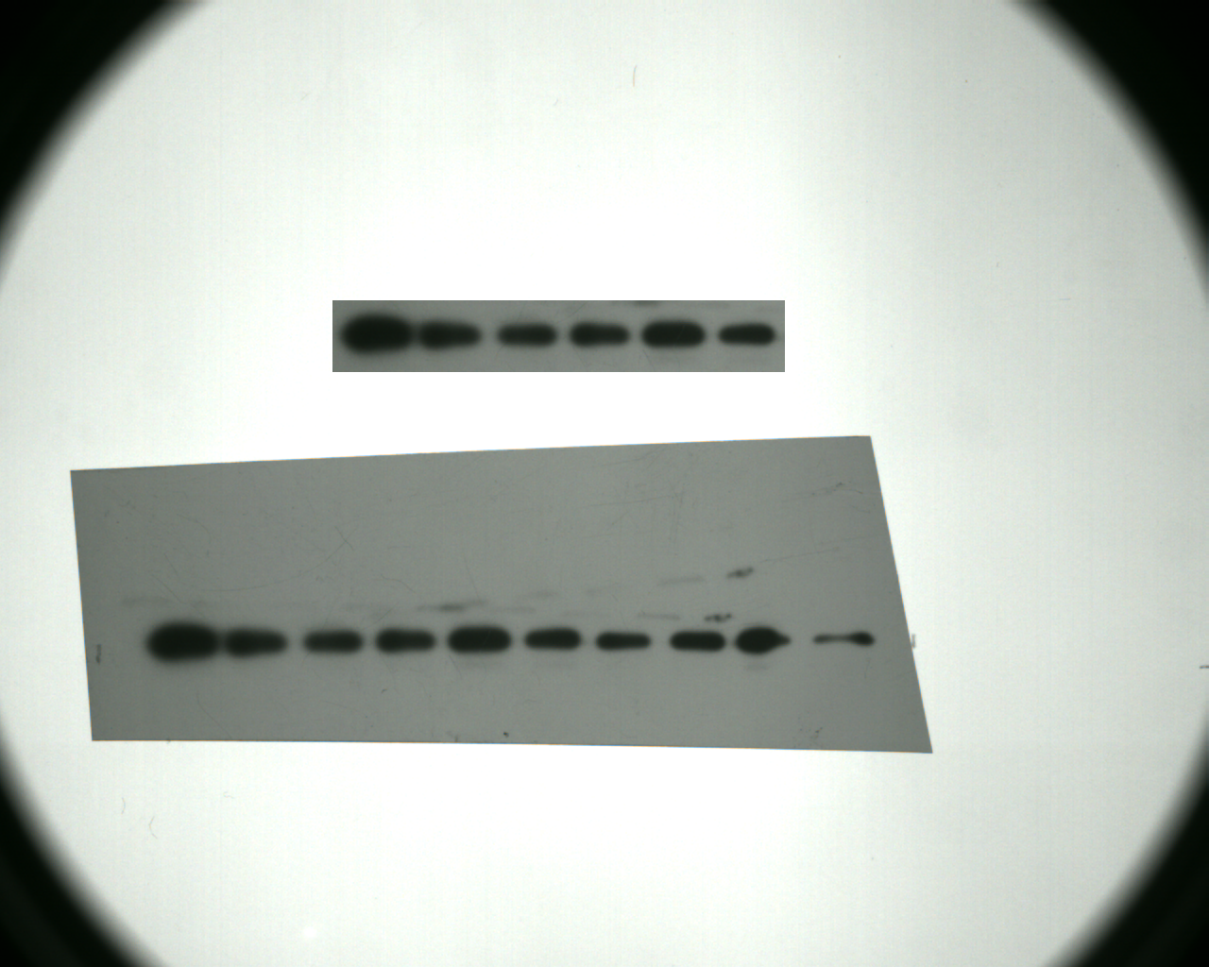


p-PI3K:


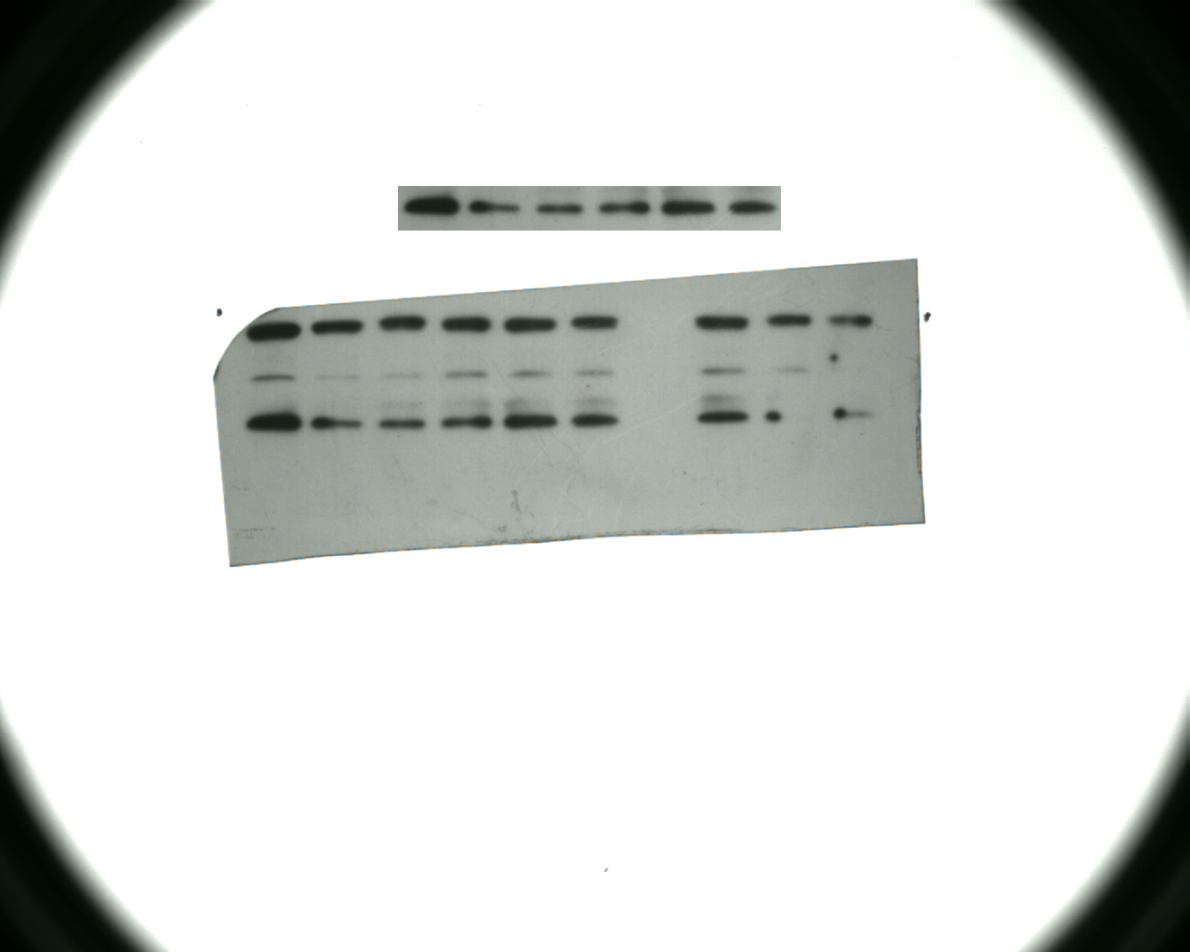


β-actin:


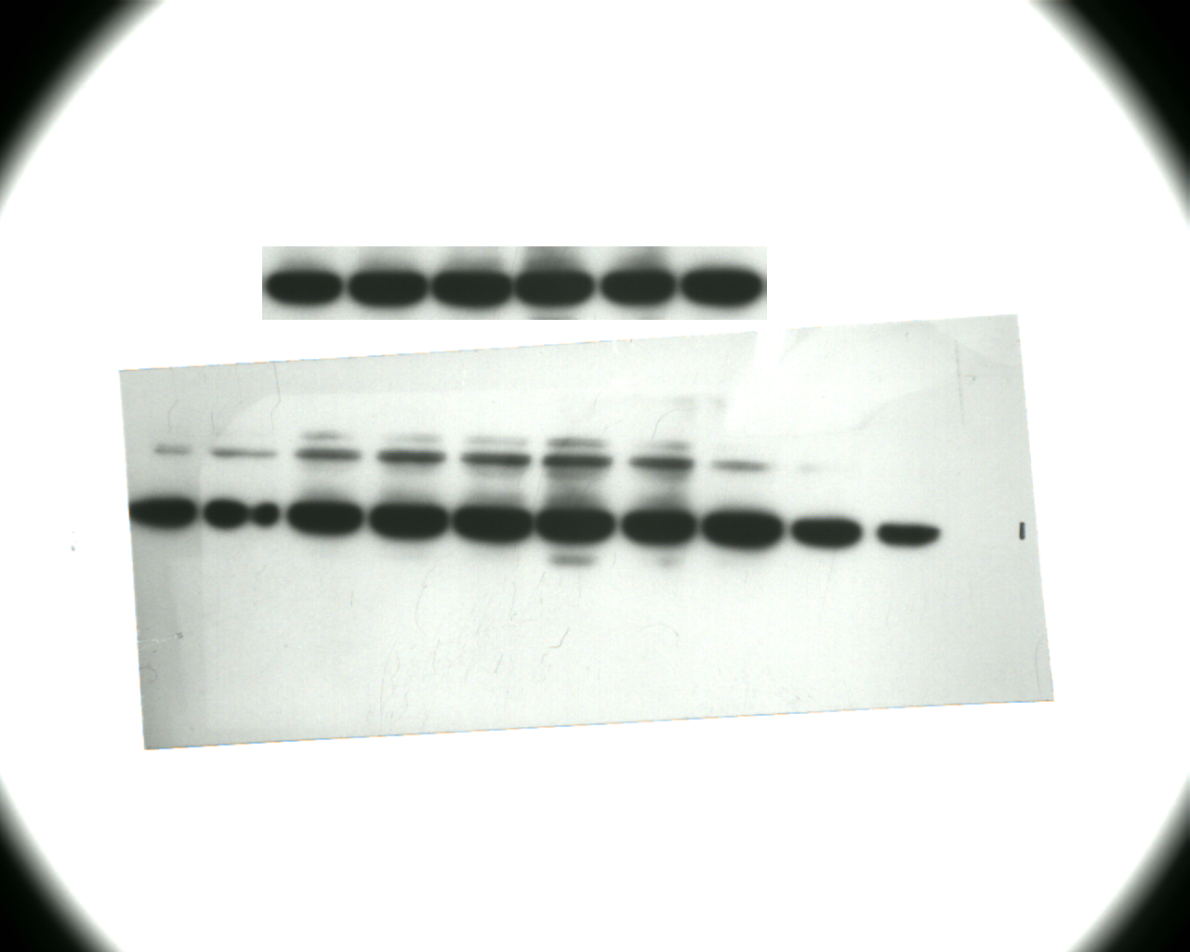


**Figure 5**

**B**

Bax:


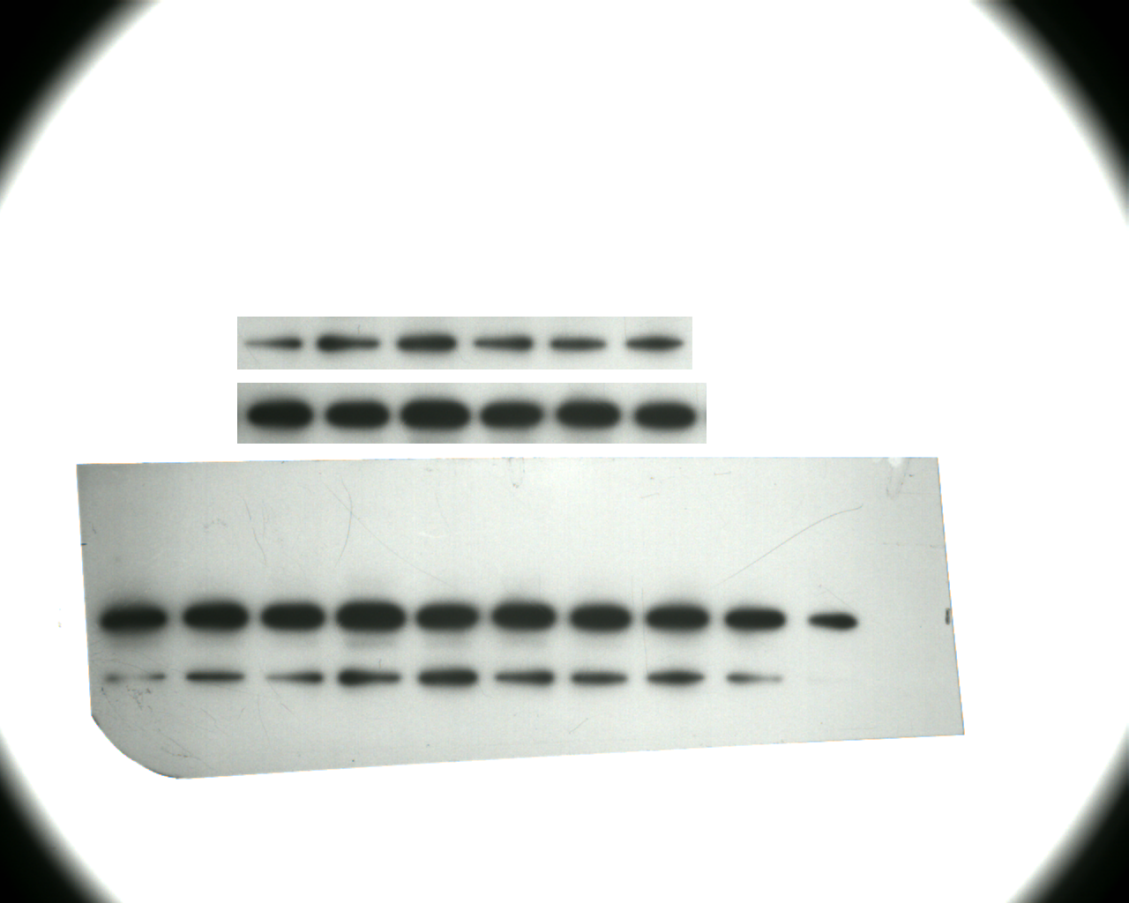


Bcl-2:


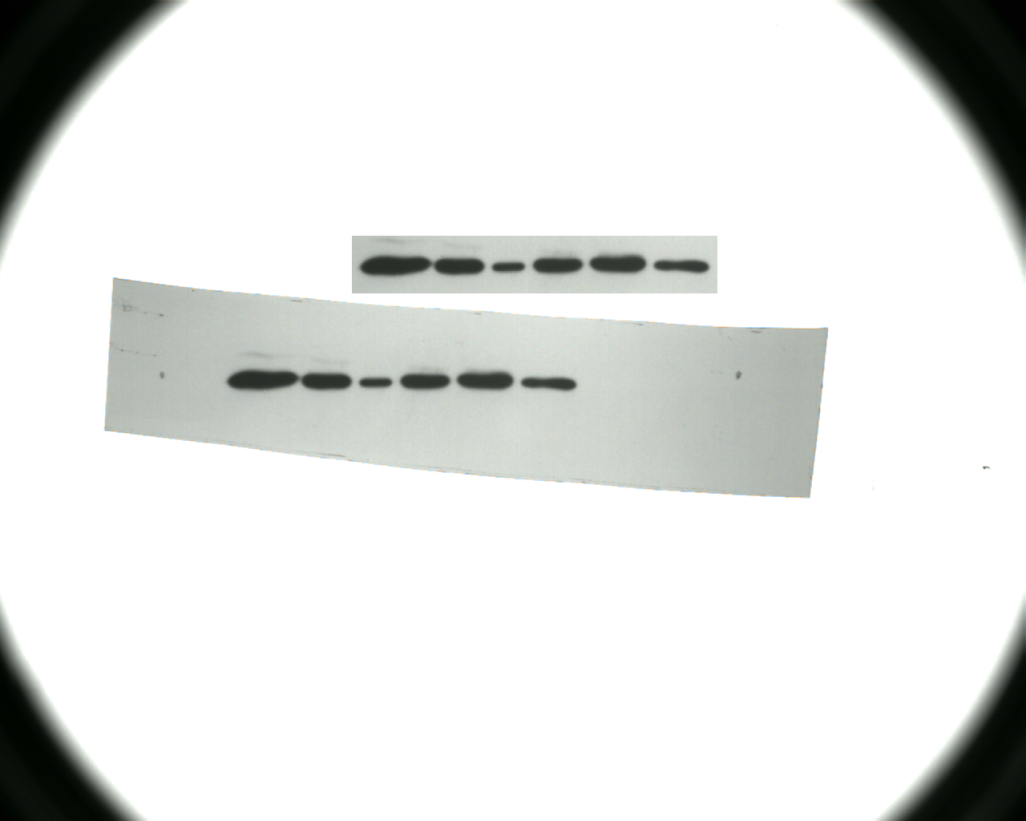


caspase-3:


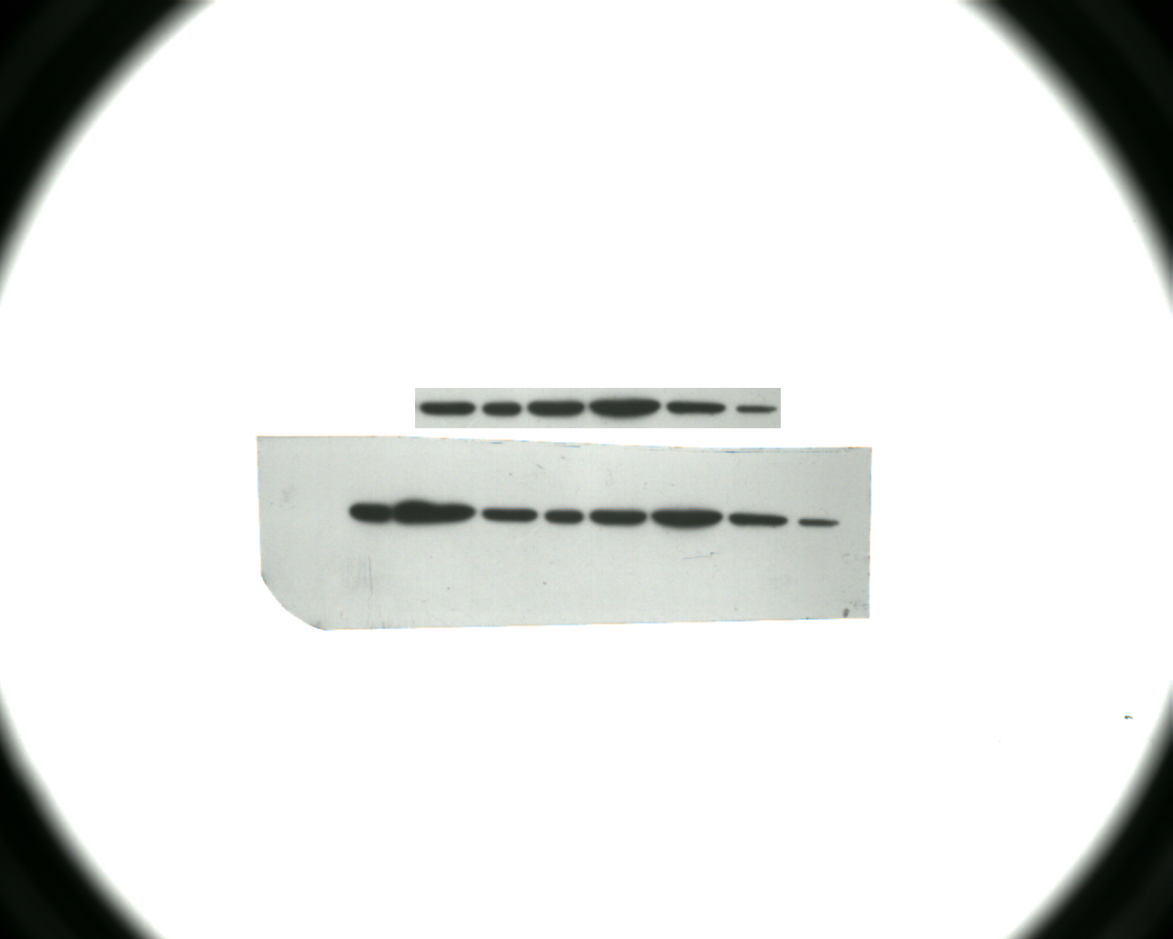


β-actin:


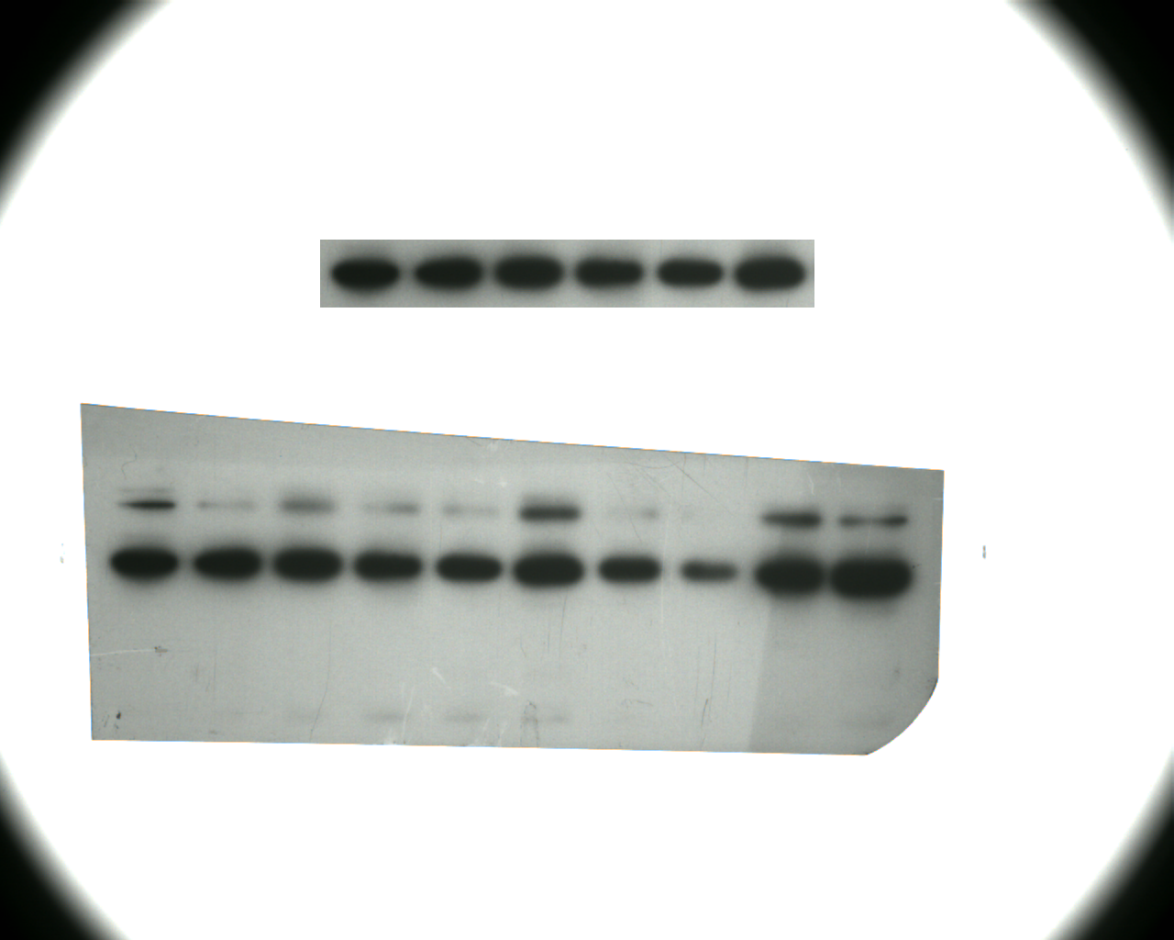


**Figure 6**

**A**

Akt:


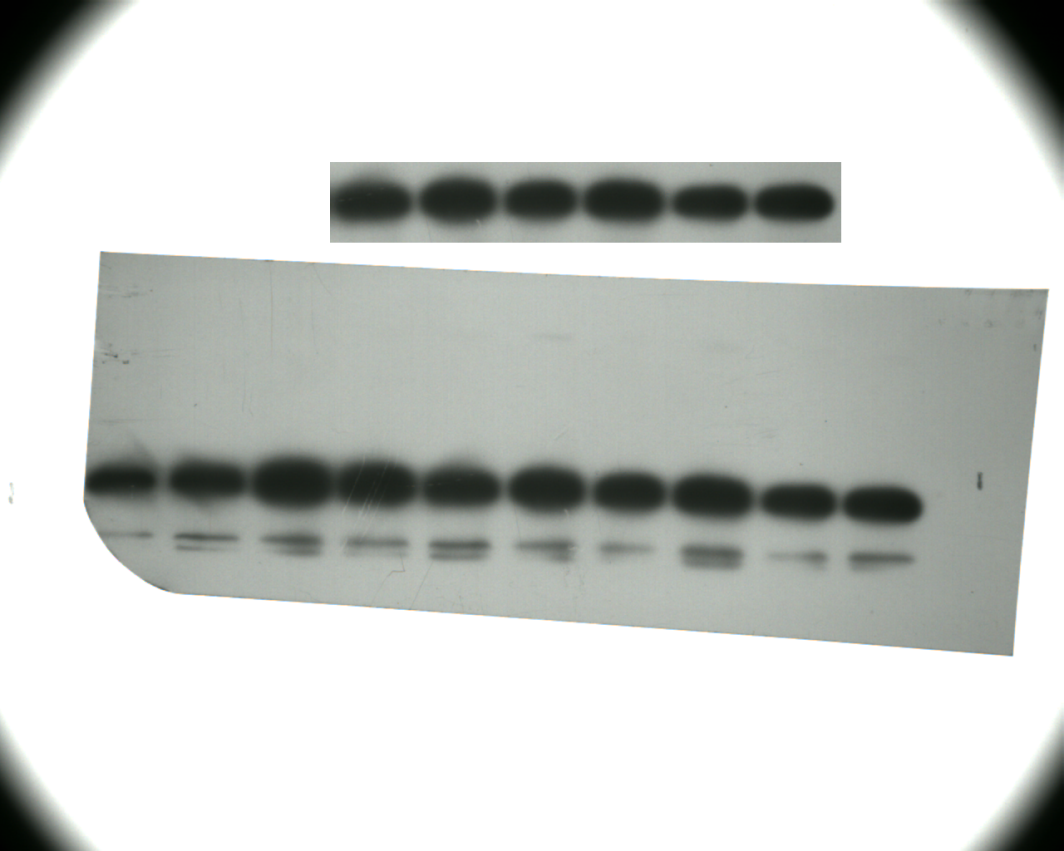


P-Akt:


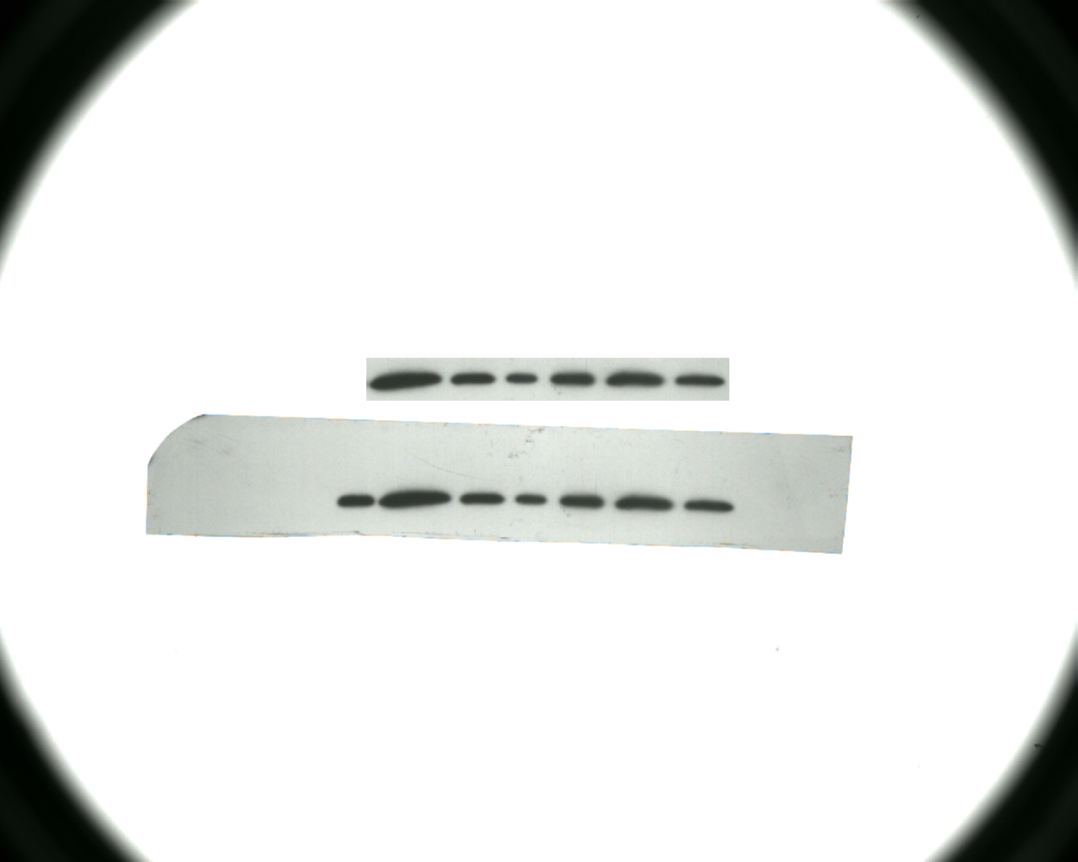


PI3K:


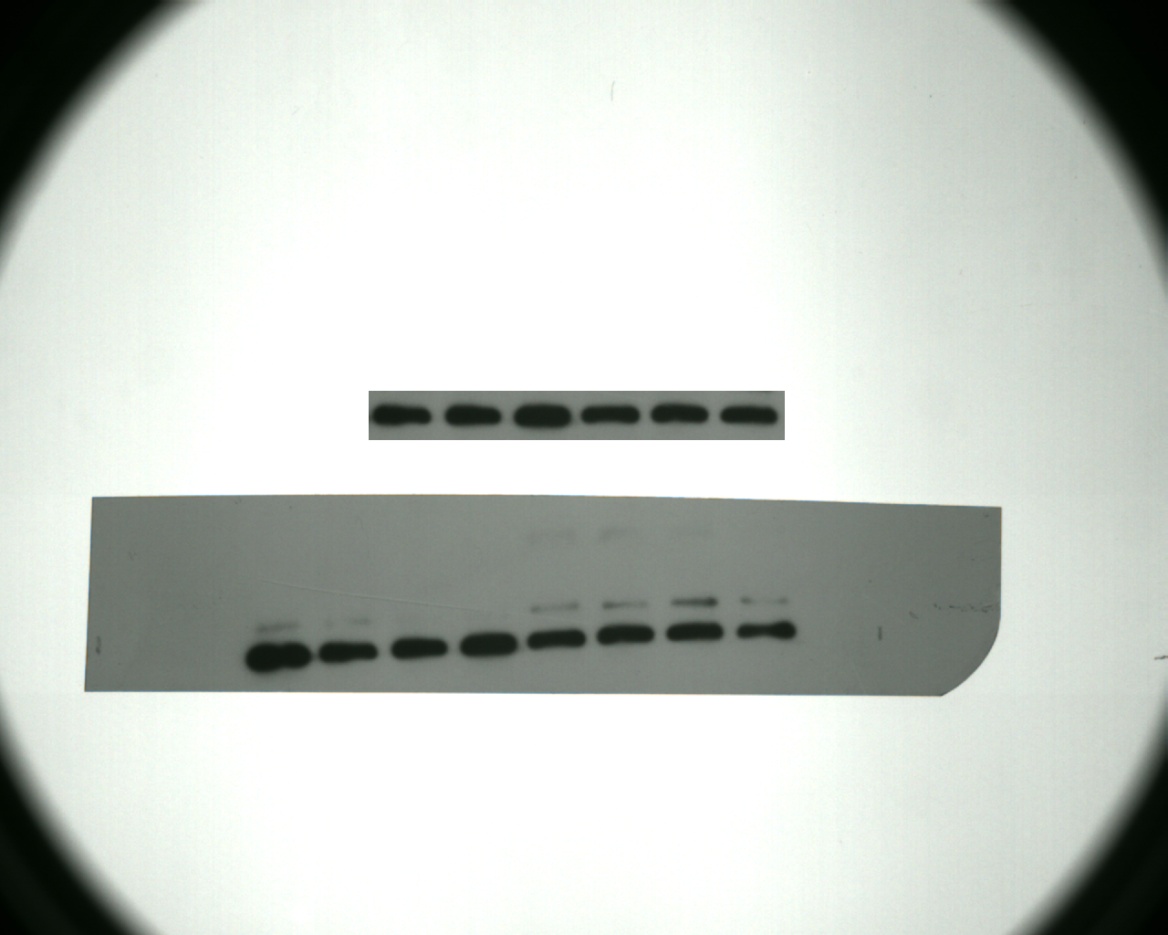


PIC3KA:


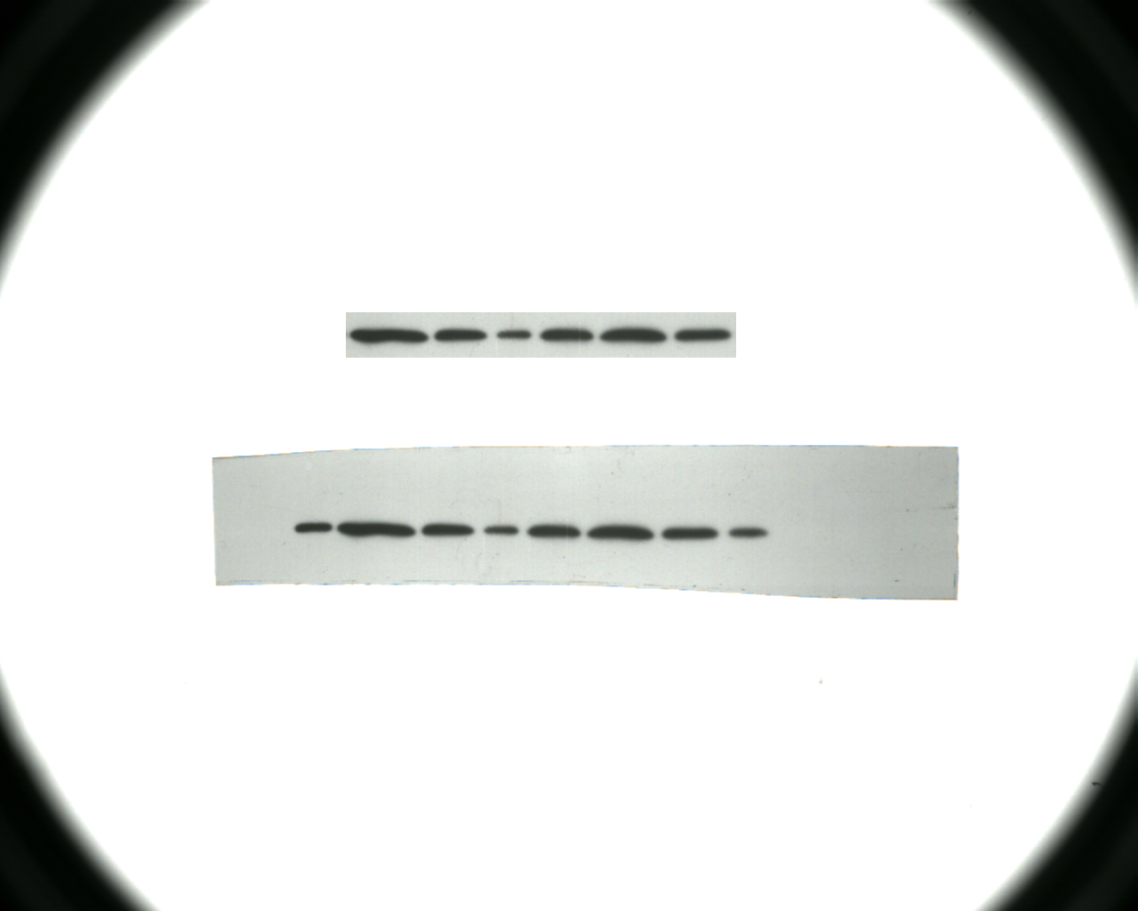


p-PI3K:


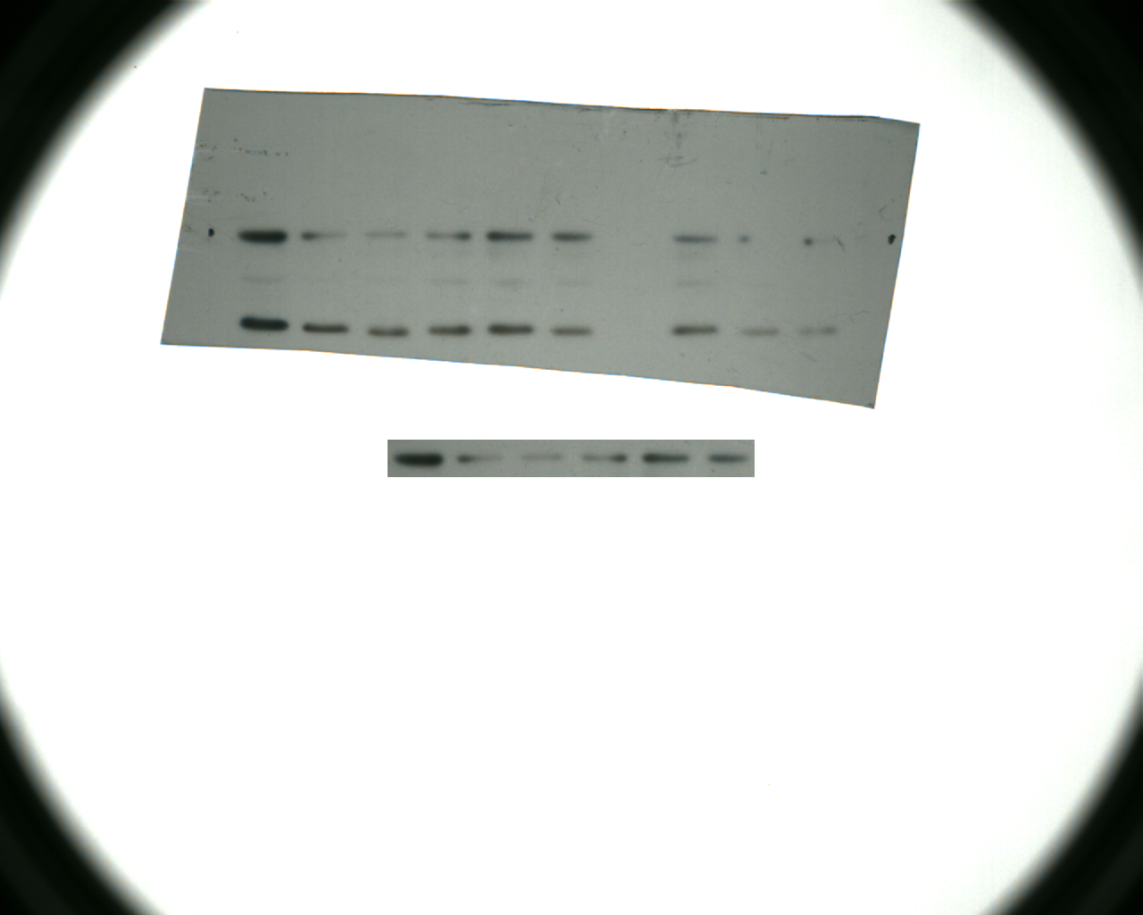


β-actin:


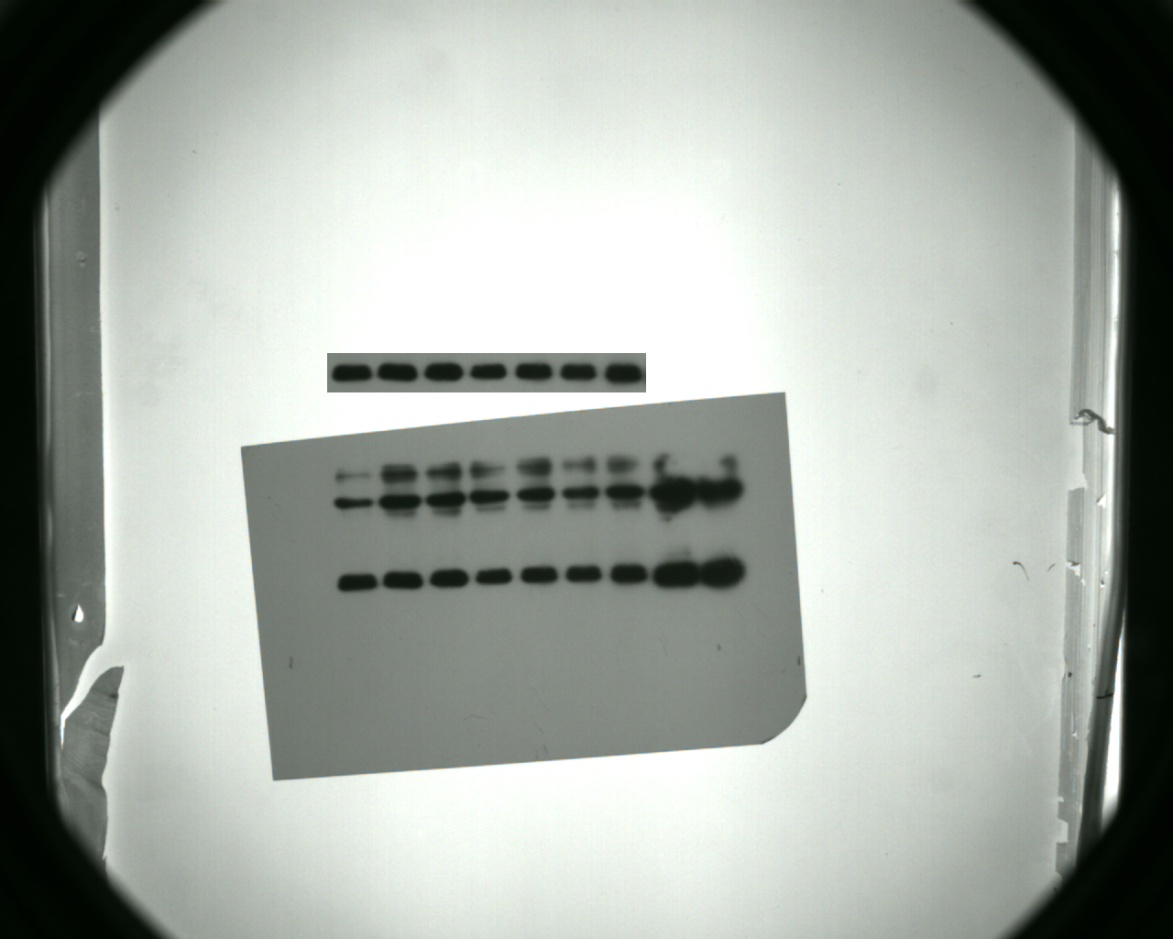

Supplement: Supplementary file 1 — Additional file 1. [file 12882_2020_1898_MOESM1_ESM.docx]
